# Noninvasive Low-Frequency Pulsed Focused Ultrasound Therapy for Rheumatoid Arthritis in Mice

**DOI:** 10.34133/research.0013

**Published:** 2022-12-19

**Authors:** Xuqiao Hu, Fei Li, Jieying Zeng, Zhenru Zhou, Zhaoyang Wang, Jing Chen, Dongyan Cao, Yifan Hong, Laixin Huang, Yongsheng Chen, Jinfeng Xu, Fajin Dong, Rongmin Yu, Hairong Zheng

**Affiliations:** ^1^ Department of Ultrasound, First Affiliated Hospital of Southern University of Science and Technology, Second Clinical Medical College of Jinan University (Shenzhen People's Hospital), Shenzhen 518020, China.; ^2^ Integrated Chinese and Western Medicine Postdoctoral Research Station, Jinan University, Guangzhou 510632, China.; ^3^ Paul C. Lauterbur Research Center for Biomedical Imaging, Institute of Biomedical and Health Engineering, Shenzhen Institutes of Advanced Technology, Chinese Academy of Sciences, 1068 Xueyuan Avenue, Shenzhen University Town, Shenzhen 518055, China.; ^4^ Shanghai Cancer Institute, Renji Hospital, School of Medicine, Shanghai Jiao Tong University, Shanghai 200032, China.; ^5^ Institute of Molecular Physiology, Institute of Cancer Research, Shenzhen Bay Laboratory, Shenzhen 518132, P.R. China.; ^6^ Department of Pharmacology, College of Pharmacy, Jinan University, 601 Huangpu Avenue West, Guangzhou 510632, China.; ^7^ Biotechnological Institute of Chinese Materia Medica, Jinan University, 601 Huangpu Avenue West, Guangzhou 510632, China.

## Abstract

Rheumatoid arthritis (RA) is a common autoimmune disease characterized by chronic and progressive inflammation of the synovium. Focused ultrasound therapy is an increasingly attractive alternative for treating RA owing to its noninvasiveness; however, it remains unclear which immune subsets respond to ultrasound stimulation. In this study, we showed that spleen-targeted low-frequency pulsed focused ultrasound (LFPFU) effectively improved the severity of arthritis in an arthritis mouse model established in DBA/1J mice. Additionally, we performed in-depth immune profiling of spleen samples from RA mice, RA mice that underwent ultrasound therapy, and healthy controls using mass cytometry along with extensive antibody panels and identified the immune composition of 14 cell populations, including CD4^+^/CD8^+^ T cells, B cells, natural killer cells, and dendritic cells. Moreover, multidimensional analysis according to cell-surface markers and phenotypes helped in identifying 4 and 5 cell subpopulations among T and myeloid cells, respectively, with 6 T cell subsets and 3 myeloid cell subsets responsive to ultrasound therapy among the 3 groups. Of these cell subsets, CD8^+^ T cell subsets showed a unique response to ultrasound stimulation in RA mice. Specifically, CD8^+^ T cells show a noticeable correlation with the degree of arthritis progression and could serve as an indicator for spleen-focused ultrasound-based therapy. Furthermore, single-cell RNA sequencing of spleen cells revealed the importance of T, B, and myeloid cell populations in the anti-inflammatory pathway. These results elucidated the unique cell subsets and transcriptome of splenic cells responsive to LFPFU and demonstrated the potential of spleen-focused ultrasound stimulation in the treatment of inflammatory diseases.

## Introduction

Rheumatoid arthritis (RA) is a systemic autoimmune disease characterized by chronic inflammation and inflammation of the synovial membrane, which eventually leads to joint destruction and dysfunction [1]. Clinically, RA is treated with a Janus kinase inhibitor that controls the symptoms and slows down the disease progression [2]. Another therapeutic strategy for treating RA is to balance inflammation by modulating the immune-regulatory signaling pathway and its associated cell subclusters, including activated T helper cells, macrophages, and regulatory B cells [3]. Apart from these approaches, implanted electrodes have been used to improve RA by modulating the cholinergic anti-inflammatory pathways that consist of the vagus nerve and the spleen circuit [4,5]. The spleen is the functional hub of this pathway, and neural innervation within the spleen is thought to affect systemic inflammation through the cholinergic anti-inflammatory pathways [6–8]. However, these approaches either result in side effects from pharmacological therapy or result from the invasiveness of implanted devices. A nonpharmacological and noninvasive therapeutic option for RA needs to be explored.

Focused ultrasound (US) therapy has attracted increasing attention owing to its noninvasive nature. Recent studies demonstrated its ability to relieve obesity-induced metabolic and inflammatory dysfunction [9] and to slow the chronic inflammatory diseases such as RA in a K/BxN model [10]. Although these findings need to be clinically verified in a larger cohort, US stimulation appears capable of reducing cytokine response to endotoxin and shows equivalent efficacy to implant-based vagus nerve stimulation [11]. Previous research using the exogenous recombination transgenic K/BxN model has optimized the parameters of spleen-focused US [10]. However, little is known about the diversity of the cell (T and myeloid cell) compartments in the enlarged spleen under inflammatory conditions. Moreover, the cell subtypes that are sensitive to US stimulation as well as the cellular markers associated with treatment efficacy remain unknown. Therefore, the design of noninvasive treatment for RA will greatly benefit from a detailed understanding of the immune cell landscape that responds to US stimulation. Furthermore, a comprehensive methodology is urgently needed to reveal the US-mediated modulation of specific cells at proteomic and transcriptomic levels.

In this study, we selectively stimulated the spleens of type II collagen-induced RA model mice (CIA model) [12] with low-frequency pulsed focused US (LFPFU) and developed multiscaled immune cell profiling in the presence or absence of LFPFU treatment. The CIA mouse model is a model of the autoantigen-mediated autoimmune disease RA that is more consistent with the characteristics of arthritis in humans and is currently the gold-standard model for evaluating RA therapy [13]. Given the utility of cytometry time-of-flight (CyTOF) mass spectrometry and single-cell RNA sequencing (scRNA-seq) analysis for revealing US-mediated changes in the proteome and transcriptome at the single-cell level [14,15], we used CyTOF to map the splenic lymphocyte immune landscape via high-dimensional analysis of splenic cell-surface markers on splenic T and myeloid cells and scRNA-seq to reveal differentially regulated genes in lymphoid and myeloid cells following US treatment to gain insights into the responsive cell subsets and genes underlying these therapeutic effects. Overall, we described the development of an advanced multiscale immune-profiling strategy for immunological mapping in the mouse spleen and elucidated the unique responsive cell subsets to LFPFU that supports its further clinical application in inflammatory diseases.

## Results and Discussion

### The effects of splenic US stimulation on arthritis severity

We used a model of inflammatory arthritis induced by the injection of type II collagen in DBA/1J mice. This RA model can achieve consistent induction of distal, symmetric polyarthritis following 2 injections of collagen into DBA/1J mice [12]. Following the second collagen injection, mice showed substantial swelling in the ankles and paws within a few days (Fig. S1A). Moreover, collection of the spleen on day 7 after the second injection revealed swelling and a larger volume relative to that observed in spleens collected from mice not receiving collagen injections (Fig. S1B). Additionally, measurement of changes in the thickness of the ankle joints indicated marked increases in paw swelling on day 5 after the second injection (Fig. S1C).

**Fig. 1.**
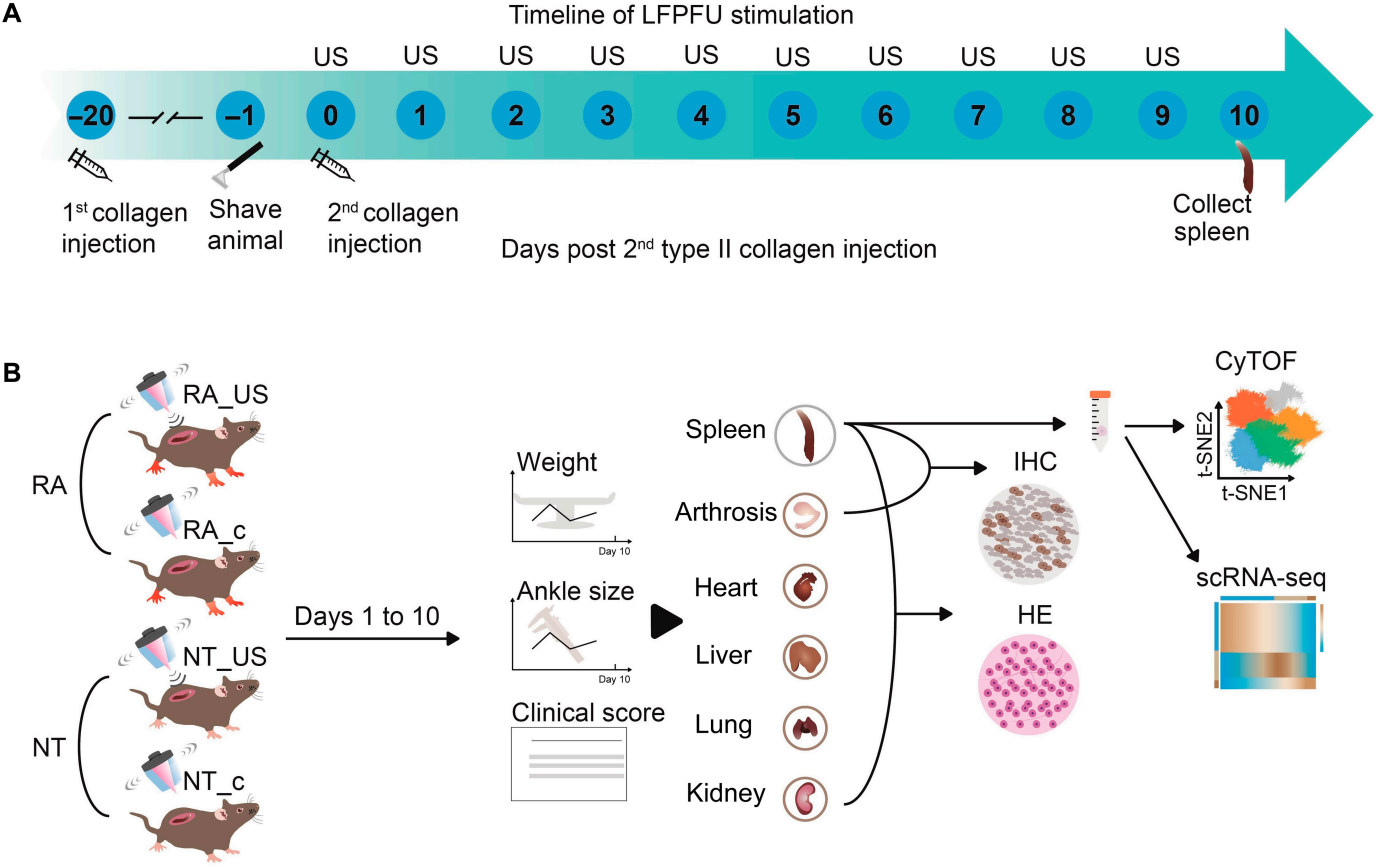
Experimental timeline and schedule of LFPFU stimulation. (A) Timeline of the experiments performed on DBA/1J mice, including 2 injections of type II collagen to establish the RA model. The second injection of collagen was conducted on day 0, and the mice were stimulated with focused US targeting the spleen on days 0 through 9. (B) Schematic representation of the experimental strategy. RA mice were stimulated with spleen-focused US (RA_US) or control US (RA_c). Mice not treated with type II collagen were set as healthy controls and also stimulated with spleen-focused US (NT_US) or control US (NT_c). On the final day of the experiments, the spleens, ankle joints, livers, lungs, hearts, and kidneys were collected for IHC and HE staining. Spleen samples were further analyzed by CyTOF and scRNA-seq.

To explore the effect of US stimulation, RA mice were anesthetized for a short period and treated with spleen-focused US (RA_US group) or control US (RA_c group) (the same setup as the stimulation group, except that no energy was delivered to the spleen), with the location, depth, and dimension of the US designed to target the spleen (Fig. S2A to C). Clinical scores used to assess the severity of arthritis were determined according to previously established methods [16,17]. We found that clinical score and ankle thickness tended to peak from days 8 to 10 after the second injection (Fig. S3A and B). Therefore, at the beginning of the second type II collagen injection, we performed US stimulation daily for 10 consecutive days (Fig. 1A). Before US stimulation, we measured the normalized sound-pressure distribution in the focal plane (Fig. S4), with each animal monitored daily to record changes in arthritis severity. On the day 10 of the experiment, the spleens, ankle joints, livers, lungs, hearts, and kidneys were collected for further assessment (Fig. 1B and Supplementary Materials). Mice not treated with type II collagen were set as healthy controls and split into 2 groups that were also stimulated with US: spleen-focused US (NT_US group) or control US (NT_c group).

**Fig. 2.**
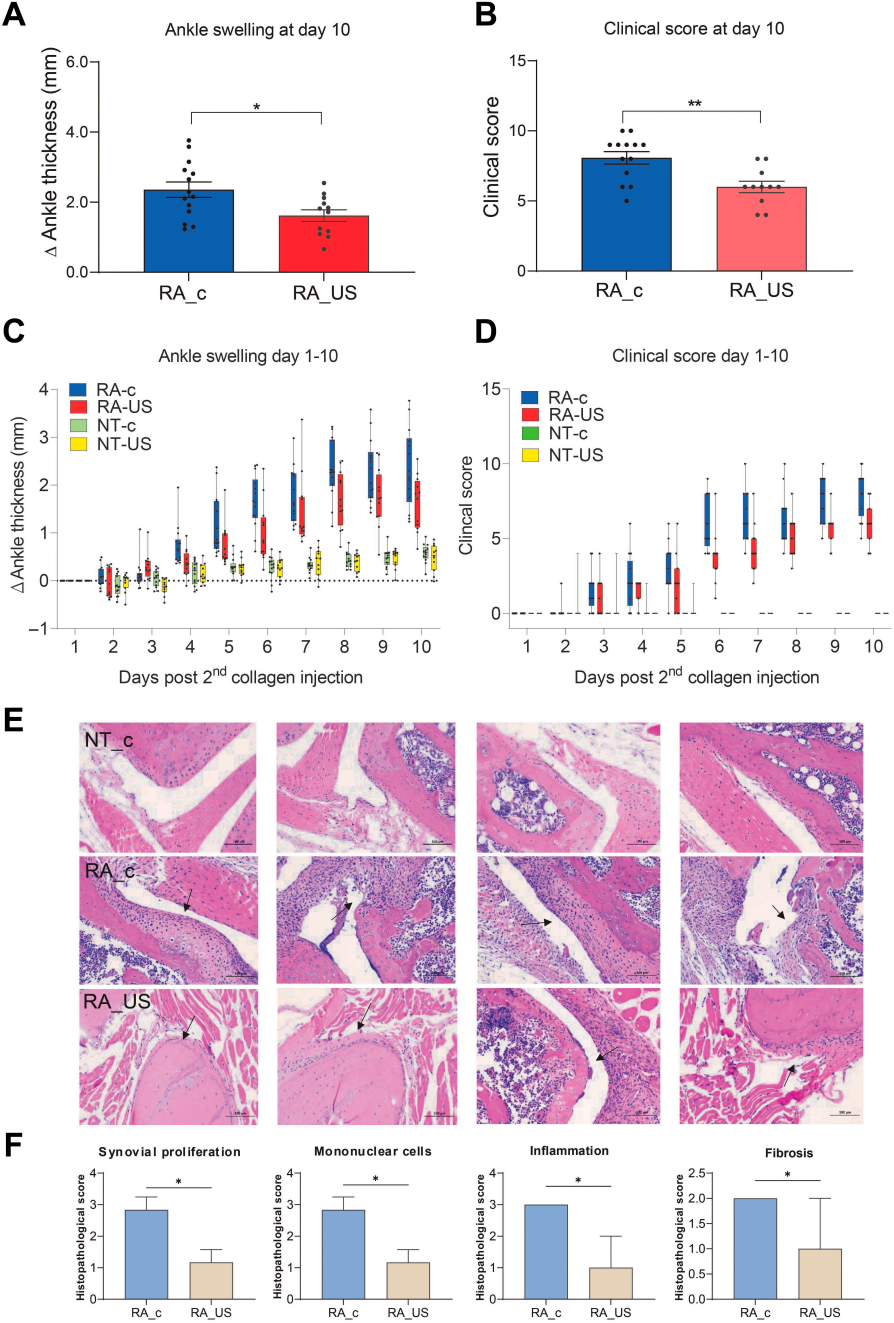
LFPFU stimulation modulates arthritis severity. Results of a 10-d arthritis experiment according to pooled data from a total of 60 mice receiving 1-MHz US focused on the spleen (350 kPa, bursts of 1 s on/5 s off for 2 min daily; duty cycle, 16.7%, RA_US). On day 10 of the experiment, changes in ankle thickness (A) and clinical scores (B) in US-stimulated RA mice (RA_US, shown in red) were significantly less than those observed in the control group (RA_C, shown in blue) (*P* = 0.015422 and *P* = 0.002573, respectively; Mann–Whitney *U* test). Pooled data for ankle swelling (C) and clinical score (D) from the full 10-d time course under the same experimental conditions with daily administration of US for 2 min on days 0 through 10 (*n* = 60). Each point indicates an individual mouse in the experiment. Data represent the mean ± standard error of the mean (SEM). *P < 0.05; **P < 0.01. (E) Histology of ankle joint inflammation. HE-stained ankle joint sections of untreated healthy mice (NT_c), unstimulated RA mice (RA_c), and US-stimulated RA mice (RA_US). Scale bar: 100 μm. (F) The magnitude of ankle joint inflammation was measured using a parameter scoring system for synovial proliferation, infiltration of the synovial membrane by mononuclear cells, inflammation, and fibrosis. Statistical analysis was performed using the Mann-Whitney *U* test to compare the scores between the 3 groups. *P < 0.05.

On day 10 after the second injection, we found that daily US stimulation of the spleen reduced the thickness of the ankle joint and the clinical score (Fig. 2A to D). Compared with unstimulated mice, Mann–Whitney tests showed that US splenic stimulation significantly improved ankle swelling (*P* = 0.015422) and clinical scores (*P* = 0.002573). Additionally, we observed changes in body weight during the course of stimulation (Fig. S5) that positively correlated with arthritis severity. On the final day of the experiment, the ankle joints were isolated for hematoxylin and eosin (HE) staining. Compared with unstimulated healthy mice (NT_c), unstimulated RA mice (RA_c) showed disorders in the bone marrow cavity network structure and obvious joint inflammation (Fig. S2E). Furthermore, the magnitude of joint inflammation was determined using a parameter scoring system for synovial proliferation, infiltration of the synovial membrane by mononuclear cells, inflammation, and fibrosis. As indicated in Fig. 2E and F, inflammation of the joints in RA mice undergoing US stimulation (RA_US) improved relative to that observed in unstimulated RA mice (RA_c). In addition, HE staining of the heart, liver, spleen, lung, and kidney did not show any side effects from splenic US stimulation compared with the differences noted in the ankle joints (Fig. S6). In a previous study [10], analysis using the K/BxN serum-transferred model demonstrated that splenic US stimulation resulted in remission of RA. Our project used a DBA/1J CIA model, which is an animal model that simulates an endogenous autoantigen-mediated autoimmune disease, and is currently the gold-standard model for studying RA [13]. Although different RA mice models have been used in these studies, there is no doubt that splenic US stimulation markedly alleviated the severity of arthritis.

### Landscape of local immunity in spleens from RA-model mice following splenic LFPFU

We then evaluated the mechanism associated with the observed reduction in joint inflammation following splenic LFPFU by investigating the immune landscape of the spleen after 10 d of US therapy via CyTOF. Mice were divided into 4 treatment groups [RA-model mice receiving US stimulation (RA_US), RA-model mice not receiving US stimulation (RA_c), healthy mice receiving US stimulation (NT_US), and healthy mice not receiving US stimulation (NT_c)], and spleen-targeted US was delivered by a 1-MHz US transducer (bursts of 1 s on/5 s off for 12 min daily). Three animals in each group (*n* = 12 total) were pooled for CyTOF analysis, with single cells from the spleen extracted separately for analysis, resulting in ~120,000 cells barcoded using unique metal isotopes before collection. The collected samples were then stained with an immune-marker panel of 42 antibodies and analyzed using CyTOF in order to identify different immune-response signatures. Single-cell suspensions of splenic samples were prepared according to method described by Cardona et al. [18].

To investigate the phenotypic diversity of splenic immune cell populations between different experimental groups, we performed unsupervised clustering based on the *k*-nearest-neighbor method [19], resulting in 44 clusters and 13 immune cell populations (Fig. S7A to C). The splenic immune landscape differed significantly between the RA and NT groups. Follicular B cells accounted for the largest proportion identified immune cells (>75% of total cells), followed by T cells and myeloid cells (Fig. S7C and D). Given that T lymphocytes and myeloid cells are critical to the pathogenesis of systemic rheumatic diseases [20,21], we reanalyzed the cells by gating the T cells and myeloid cells and performing a second round of clustering.

**Fig. 3.**
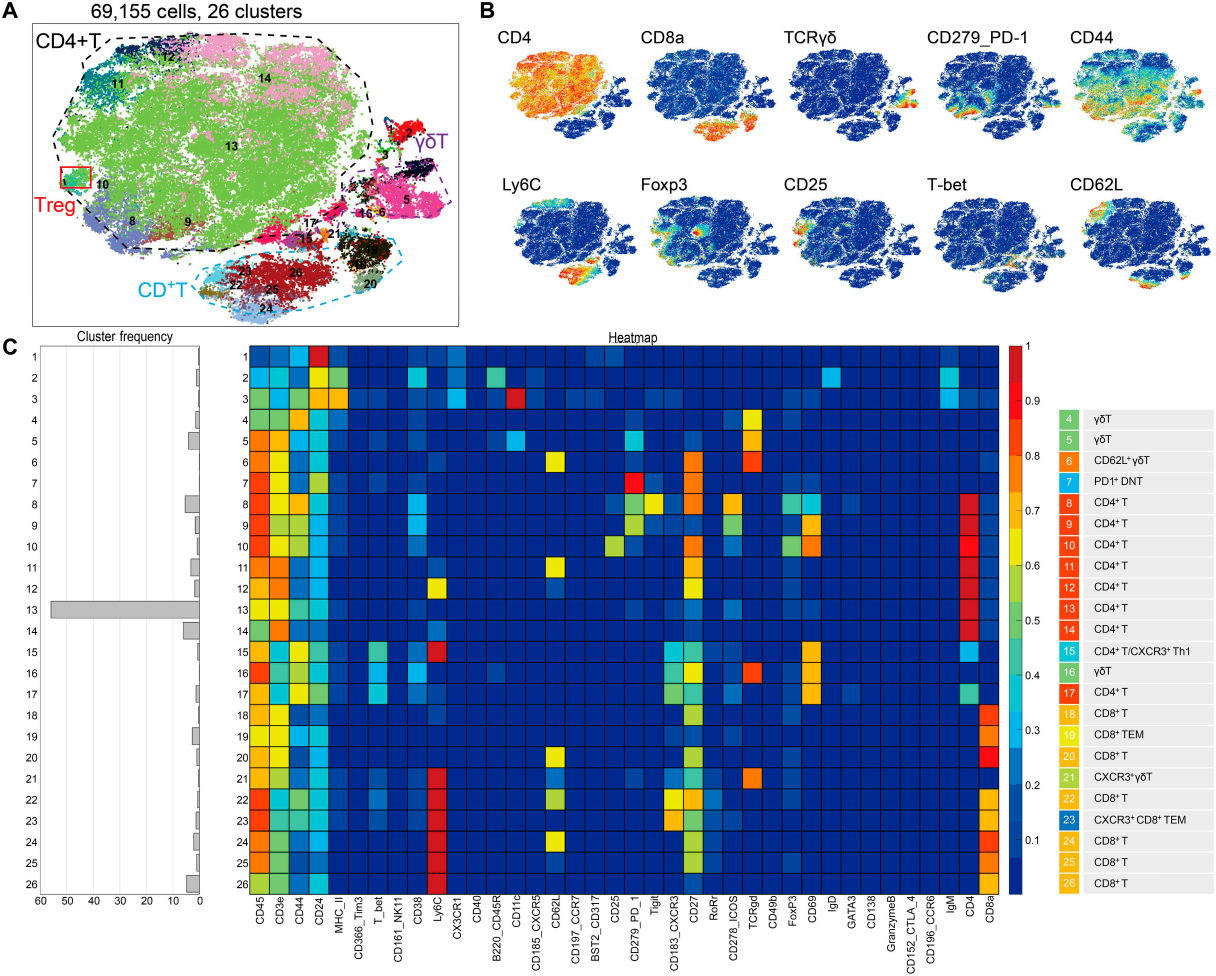
The detailed heterogeneity in the splenic T cells of RA models. (A) t-SNE plot of 26 identified clusters (B) color-coded according to the expression of 10 marker genes for 4 main T cell subtypes. (C) Heatmap showing the differential expression of 42 markers among the 26 clusters. Certain clusters were identified as known cell types according to typically expressed markers.

**Fig. 4.**
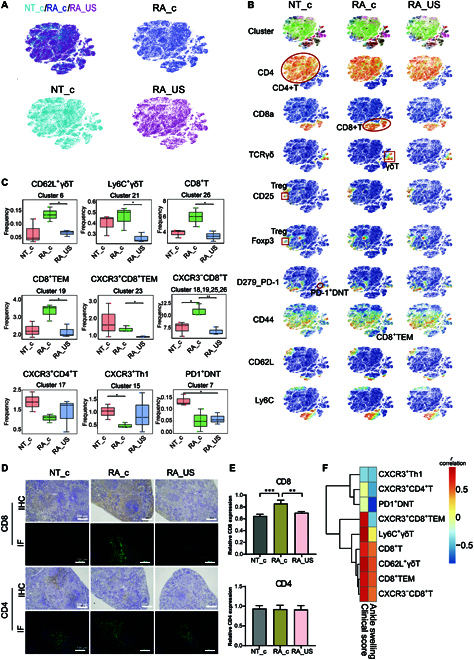
Subcluster differences according to T cell distribution. (A) Difference in T cell subsets among the 3 groups. (B) t-SNE plots color-coded according to the expression of marker genes based on T cell subset. (C) Frequencies of 9 main types of immune cells in the 3 groups. *P < 0.05; **P < 0.01; ***P < 0.001. (D) IHC and IF assays of CD8 and CD4 expression in the spleen from of untreated healthy mice (NT_c), unstimulated RA mice (RA_c), and US-stimulated RA mice (RA_US). Scale bar: 200 μm for IHC assay and 100 μm for IF assay. (E) Relative CD4 and CD8 expression of spleen in different groups. Data are presented as the mean ± SEM (*n* = 3). **P < 0.05, ***P < 0.001. (F) Correlation analysis of the populations of the 9 cell subsets and arthritis progression evaluation in terms of ankle swelling and clinical score. *r* represents the Pearson correlation coefficient.

A t-distributed stochastic neighbor embedding (t-SNE) map of the diverse T and myeloid cell populations is shown in Fig. 3. T cells were isolated according to cell-surface marker expression (Fig. 3B and C, and Fig. S8), resulting in analysis of >69,155 single cells from 9 splenic samples and identification of 26 clusters. Initially, 4 main populations were identified among T cells (Fig. 4A and B), including populations of CD4^+^ T cells, regulatory T cells (Tregs), γδT cells, and CD8^+^ T cells (Fig. 3). Among them, Treg deficiency is reportedly critical for the development of autoimmune diseases [21]. For CD8^+^ T cells, some autoreactive multifunctional CD8^+^ T cells recognize self-antigens with high affinity and counter-regulate Treg levels in patients with RA [22]. Therefore, these subclusters of T cells might be associated with improvements in RA during US treatment. To determine the mechanism by which US stimulation of the spleen improves RA and the possible targeted cell populations, we compared the proportions of immune cells in each cluster among the NT_c, RA_US, and RA_c groups (Fig. 4A to C).

Figure 4C shows reductions in γδT cells after US stimulation, including the CD62L^+^ and Ly6C^+^ γδT subsets. CD62L reportedly acts as an adhesion molecule that regulates leukocyte migration to chronic inflammatory tissues [23], and Ly6C is mainly expressed in effector T cells and regulates the immune function of peripheral inflammation [24,25]. γδT cells exert proinflammatory effects, with reduced γδT cell levels reportedly correlated with improved arthritis symptoms in humans [26]. The reduction in these subclusters suggests that these T cell subsets were responsive to splenic US stimulation. Additionally, levels of CD8^+^ T cells, including CD8^+^ T effector memory (TEM) cells and C–X–C motif chemokine receptor 3 (CXCR3)^+^CD8^+^ TEM cells and their subclusters, were attenuated following splenic US treatment. Specifically, the number of CD8^+^ TEM cells decreased in the RA_US group, especially CXCR3^+^CD8^+^TEM subsets (Fig. 4C), suggesting that this subset was responsive to US. CD8^+^ TEM cells present a phenotype similar to that of effector cells, which exhibit rapid effector functions and can easily differentiate into effector T cells that secrete an interferon-γ and are highly cytotoxic [27]. CXCR3 is closely associated with T cell distribution, migration, and function, and we observed attenuated levels of the CXCR3^−^CD8^+^ T cell subset upon recovery of RA-model mice following US stimulation (Fig. 4C), suggesting the importance of this change to the recovery process. Additionally, compared with healthy mice, levels of CXCR3^+^CD4^+^ T and CXCR3^+^ T helper 1 subsets were attenuated in RA-model mice, with no changes in their levels observed following splenic US stimulation. Similarly, we observed no changes in programmed cell death (PD) 1-related subsets (Fig. 4C), indicating that these subclusters were nonresponsive to US. Collectively, unique CD8^+^ T cell clusters respond to US stimulation in CIA model mice.

Interestingly, the number of CD8^+^ T cells appear to be correlated with the degree of arthritis progression. CD4^+^ T cells have long been considered one of the major immune cells that mediate the pathogenesis of RA [28]. In contrast, the role of CD8^+^ T cells in collagen-induced arthritis is poorly studied and controversial [29]. A recent study reported that CD8^+^ T cells are potentially key mediators of RA pathogenesis [30]. Additionally, CD8^+^ T cells (e.g., CD8^+^CD25^+^ T cells) were upregulated in peripheral blood mononuclear cells and synovial fluid in RA patients [31]. Consistent with the clinical studies, the number of CD8^+^ T cells was elevated in RA mice in our study, which used a CIA mouse model, compared to healthy mice. After splenic US stimulation, the number of CD8^+^ T cells was reduced and arthritis was alleviated. To further confirm the role of CD8^+^ T cells in RA pathogenesis, the in situ expression of CD8 in the spleen were further assessed by immunohistochemical (IHC) and immunofluorescence (IF) assays (Fig. 4D and E). The semiquantified results of IHC assay revealed that the expression of CD8 in spleen was dramatically elevated in RA mice (RA_c) when compared with that in healthy mice (NT_c), and this level was attenuated in RA mice after US treatment (RA_US). Therefore, the decrease in disease severity upon US stimulation could be explained by the changes in CD8^+^ T cell population in the spleen. To confirm the relationship between the number of cells and the progress of arthritis, we conducted a correlation analysis between cell population and degree of arthritis severity. As shown in Fig. 4F, a noticeable positive correlation was observed between the numbers of CD8^+^ T cells and its subsets (e.g., CD8^+^ TEM and CXCR3^−^CD8^+^ T cells) with either clinical score or ankle swelling. The Pearson correlation coefficients of CD8^+^ T, CD8^+^ TEM, and CXCR3^−^CD8^+^ T cells with clinical score were 0.70, 0.78, and 0.82, respectively. The Pearson correlation coefficients of these 3 CD8^+^ T subsets with ankle swelling were 0.49, 0.53, and 0.48, respectively. Thus, the number of splenic CD8^+^ T cells is associated with the degree of arthritis progression and could serve as an indicator for assessing US-based LFPFU therapy.

**Fig. 5.**
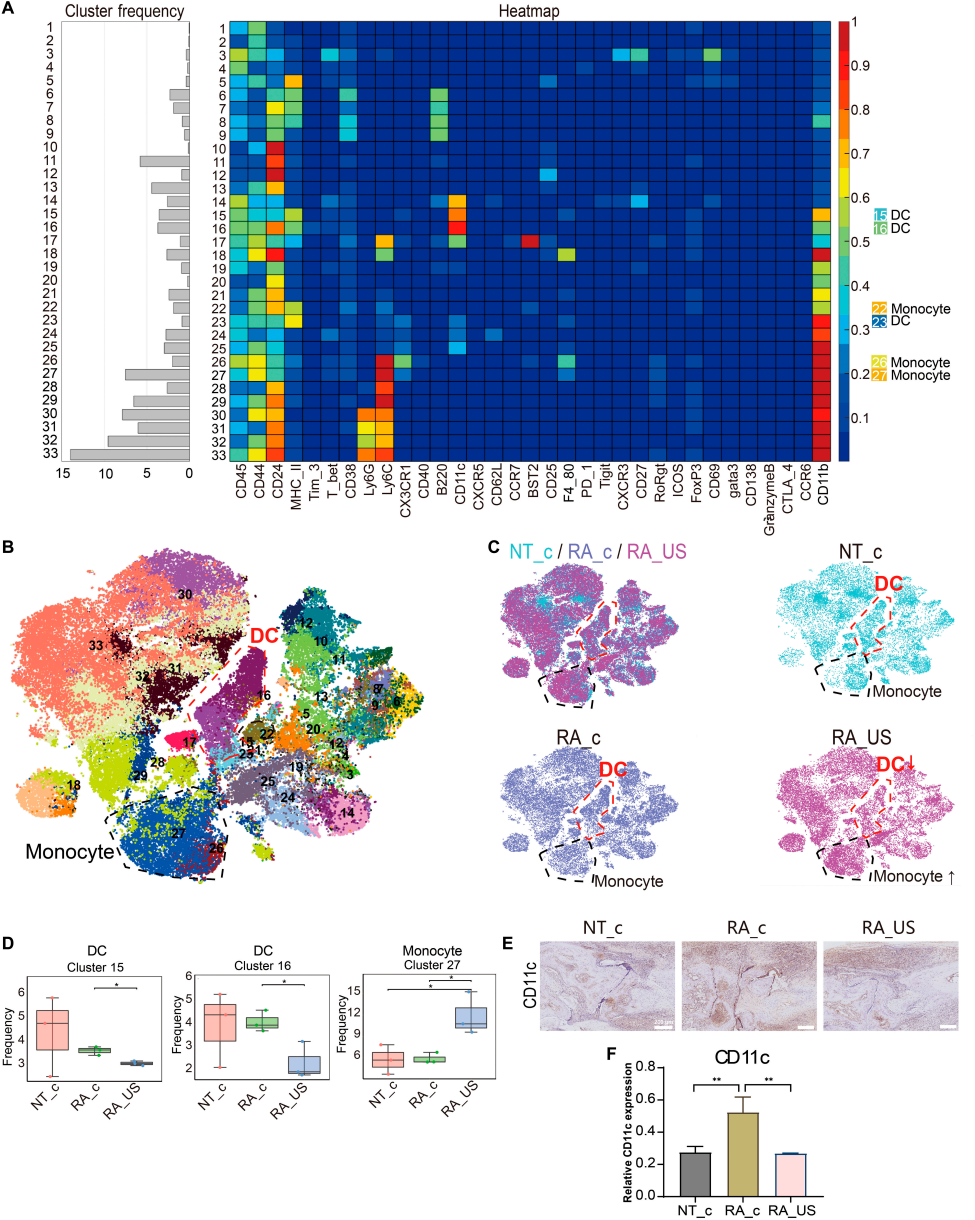
Differences subclusters of splenic myeloid cells. (A) Heatmap showing the differential expression of 42 immune cell markers among (B) 33 identified cell clusters according to a t-SNE plot. (C) Differences in the landscape of myeloid cells among the identified groups. (D) Frequencies of 6 clusters of myeloid cells among the groups. *P < 0.05. (E) IHC assay of CD11c expression in the ankle joints from of untreated healthy mice (NT_c), unstimulated RA mice (RA_c), and US-stimulated RA mice (RA_US). Scale bar: 200 μm. (F) Relative CD11c expression of ankle joint in different groups. Data are presented as the mean ± SEM (*n* = 6). **P < 0.01.

Myeloid cells were gated according to cell-surface marker (Fig. 5A and Fig. S9), 33 clusters including dendritic cells (DCs) and monocytes were discovered (Fig. 5A and B). Among RA-model mice, the number of splenic DCs (MHCII^+^CD11c^+^CD11b^+^CD44^+^CD24^+^) cells decreased after US stimulation (Fig. 5C and D). As indicated in Fig. 5D, DC subsets in RA mice with or without US stimulation were lower than those in healthy mice, while the number of monocytes was markedly increased after US stimulation in RA mice (Fig. 5C and D). DCs are involved in RA pathogenesis and accumulate in the synovial membrane, where they produce large amounts of proinflammatory factors [32]. DCs have strong T cell activation functions and play an important role in the generation and maintenance of synovial inflammatory response [33]. The synovium of RA patients contains a large number of DC with high T cell-stimulating capacity, which produces proinflammatory factors to aggravate the inflammatory response of joints [34]. During T cell activation, DCs serve as antigen-presenting cells, on which pathogenic peptides are identified by the CD8^+^T cells [35]. Furthermore, CD8^+^T seems to crosstalk with DCs, a study reports that CD8^+^NKT-like cells suppress T-cell responses through the elimination of DCs [36]. Thus, CD8^+^T cell subsets also function as suppressive cells to regulate the immune response through killing DCs. Therefore, the diversity and adjustability of DCs in immune response make DCs an effective target for specific treatment of RA. In our study, we also observe the responsiveness of the DCs after US stimulation. The number of DCs and CD8^+^T cells decreased markedly in RA mice that underwent US stimulation. To further confirm whether the DCs change in the ankle joint, we assessed the in situ expression level of using the CD11c marker through IHC in ankle joint tissues (*n* = 6). The semiquantified results of the immunohistochemistry assay revealed that the expression of CD11c in the ankle joint of control arthritic mice (RA_c) was elevated compared with that in control healthy mice (NT_c); the expression of CD11c was attenuated after US treatment (RA_US) (Fig. 5E and F). Therefore, the decrease in disease severity upon US stimulation could be partially explained by the changes in DC population in the ankle joints.

Regardless of the DCs, the CD8^+^T cells were thought to contribute to the development of RA. CD8^+^T cells are abundant in the synovium of RA [37], and in the lymph nodes and peripheral blood mononuclear cells of patients with early RA [38]; their abundance is closely correlated with RA activity [39]. Typically, in active RA, CD8^+^T cells with an exacerbated effector and activating phenotype were detected in synovium [40]. As a center immune organ, CD8^+^T cells in spleen may contribute to the pathophysiology of RA. Neuromodulation through vagus can regulate the pathophysiology of RA, as it affects spleen immune function [41]. Noninvasive US energy transfer to their abdomen reduces the inflammatory response and tissue damage, and these anti-inflammatory effects are mediated by the spleen [42]. In our study, we observed an alleviation in arthritis and a reduction in CD8^+^T cells in the spleen after US stimulation, which was consistent with the results of previous studies [10]. We hypothesize that splenic US stimulation relieves RA by attenuating the splenic CD8^+^T cells, eliminating DCs, and then exerting an anti-inflammatory effect. This study provides new insight into the neuromodulation of RA by US and may provide a nonpharmaceutical and noninvasive therapy mode for RA treatment.

To investigate the interference caused by US itself, US stimulation of the spleen in healthy mice was conducted for 10 d (*n* = 6). We observed that US stimulation resulted in different responses between healthy and RA mice, indicating that US stimulation has a unique effect on RA mice. Heatmaps of T cells and myeloid cell subclusters are shown in Fig. S10A and B. Single nuclear cells from the spleen were analyzed by CyTOF, and T and myeloid cells were extracted and analyzed separately. Approximately 60,000 single cells from 6 splenic samples were analyzed, and 28 clusters were isolated. In T cell subclusters, CD27^+^ PD1^+^ γδT and CD11c^+^ γδT subsets decreased after US stimulation (Fig. S10C), which was different from the effect of US on RA mice. The cluster frequencies were also compared in myeloid cells, and macrophage and neutrophil subsets increased after US stimulation in healthy mice (Fig. S10D). As previously reported, these subclusters were not observed in mice with RA (Figs. 4 and 5).

### High-dimensional single-cell transcriptomics analysis reveals response to US stimulation of the spleen in the RA model

**Fig. 6.**
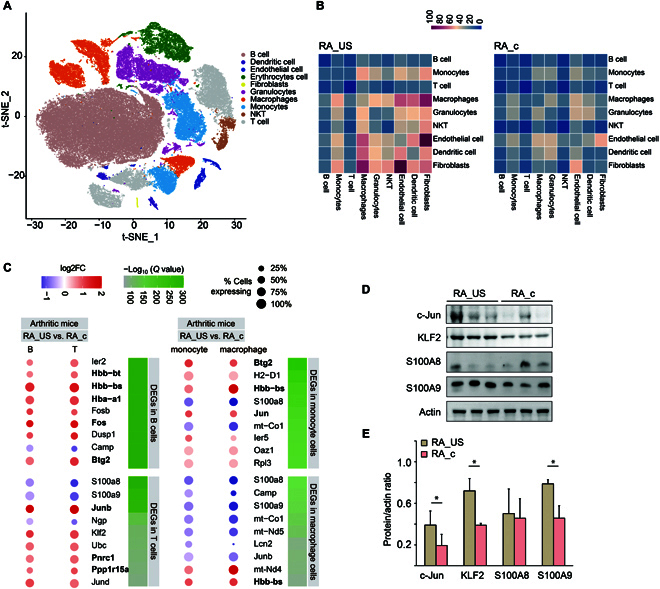
scRNA-seq analysis of 107,559 splenic mouse cells. (A) t-SNE dimensional reduction of 107,559 single splenic cells that were profiled. Each point represents an individual cell and is color-coded according to its assigned cell type. (B) Heatmap showing the interactions between cell types in the spleen obtained with CellPhoneDB in both the RA_US and RA_c groups. Rose-red and blue represent a high and low number of interactions, respectively. (C) The dot plot depicts the statistically DEGs in T cells, B cells, macrophages, and monocytes from RA-model mice relative to controls. The size of each circle represents the percentage of cells within each cell type and with the gene listed along with the color of each circle, which represents the log_2_ fold change of average scaled expression in the RA_US relative to the RA_c group. The −log_10_ (*Q* value) for each gene in all cells is shown in shades of green. All DEGs listed had a *Q* value <9.98 × 10^−23^ according to Benjamini–Hochberg tests. (D) Splenic US stimulation promotes the upregulation of *c-Jun*, *KLF2*, and *S100A9*. Representative western blot (D) and relative protein expression (E) in spleen tissue from the RA_US and RA_c groups normalized against actin. *P < 0.05.

The data suggested the unique efficacy of spleen-focused US stimulation in RA mice and that some subclusters may contribute to this effect. Therefore, we investigated the transcriptome of inflammation reduction observed with US stimulation using scRNA-seq in spleen samples from RA_US, RA_c, NT_US, and NT_c mice after 10 d of US treatment. Two mice from each group were sacrificed (*n* = 8). For scRNA-seq analysis, single nuclear cells from the spleen were extracted separately, and 107,559 single cells were analyzed by scRNA-seq. We identified 10 major cell clusters and characterized the cellular properties of T cells, B cells, macrophages, monocytes, DCs, endothelial cells, erythrocytes cells, fibroblasts, granulocytes, and natural killer (NK) cells (Fig. 6A). Correlation analysis was further conducted using the average expression of 41 protein markers or corresponding genes in different cell types to determine the correlations between the results of the CyTOF and scRNA analyses of the cell types. As shown in Fig. S11, there was a noticeable correlation between the CyTOF and scRNA results. To understand the effects of US stimulation on myeloid cells, we performed pseudotime analysis of all 28,675 myeloid cells [43] according to group and cell types (Fig. S12). Interestingly, myeloid cells in each group showed similar trajectories among the 4 groups, indicating that RA induction or US stimulation had little effect on myeloid cell differentiation. To evaluate differences in immune response, we constructed a cell–cell-interaction network via known ligand–receptor pairs [44] within the 10 identified clusters. Ligand–receptor pairs (*P* < 0.05) were considered important interactions between the 2 cell types. As shown in Fig. 6B and Fig. S13A, in the NT_c group, most intercellular interactions occurred between fibroblasts and endothelial cells, whereas in the RA group, this interaction was dramatically reduced from 100 to 50 interactions along with other interactions between fibroblasts, macrophages, and monocytes. However, in the RA_US group, the fibroblast–endothelial cell and fibroblast–granulocyte interactions were restored to normal levels in the NT_c group. One of the markedly enriched ligand–receptor pairs was BMPR1A_BMPR2–BMP2 (Fig. S14A). BMP2 reportedly plays an essential role in osteogenesis and induces cartilage and bone formation [45]. Additionally, we identified 10 special ligand–receptor pairs between fibroblasts and macrophages/monocytes (Fig. S14B and C). Of the ligands involved in immune and inflammatory responses, the colony-stimulating factor-1 receptor–interleukin 34 pair was markedly enriched. Interleukin 34, a cytokine, reportedly plays an important role in regulating osteoblast proliferation, differentiation, and bone resorption [46]. Another ligand–receptor pair [cell adhesion molecule 1 (CADM1)–CADM1] was also highly enriched in the RA_US group. CADM1 is a unique cadmium-dependent protein that promotes NK cell cytotoxicity and interferon-γ secretion by CD8^+^ T cells [47]. These results suggest the role of US stimulation in treating RA by restoring the cell–cell interactions that were absent in RA_c mice. Notably, only minor changes in intracellular interactions were observed between NT_c and NT_US mice, which indicates that US stimulation has little interference in immune cell interactions in normal mice (Fig. S13B).

We then explored differentially expressed genes (DEGs) between splenic cells in RA-model mice upon US stimulation. Among the RA mice, the DEGs in T and B cells possibly attributed to US stimulation are shown in Fig. 6C (left). DEGs in healthy mice subjected to no or some stimulations were identified as controls (Fig. S15). In the T cells of RA mice, we identified 9 DEGs with *Q* values <1.72 × 10^−92^ (ranging from Q = 3 × 10−^301^ to 1.72 × 10^−92^) as compared with the US-stimulated RA_US group versus no-US-stimulated RA_c mice (Fig. 6C); however, *S100a8*, *S100a9*, and *Ngp* were upregulated with US stimulation. Similarly, we found 9 DEGs (RA_US versus RA_c) in B cells in RA mice (*Q* values < 9.9 × 10^−297^), and all but *Camp* was upregulated with US stimulation (Fig. 6C). Of note, most DEGs found in T cells of RA mice were not differentially expressed in healthy mice (except *Junb*, Pnrc1, and *Ppp1r15a*), indicating a unique effect of US stimulation on RA mice. Additionally, 14 of 18 DEGs were upregulated in both T and B cells of RA mice, demonstrating that US induces overlapping effects in both cell types. We observed that the genes in T and B cells were notably regulated upon spleen-focused US therapy, which is consistent with the literature showing that these cell types are involved in splenic anti-inflammatory pathways [48]. These findings suggest that the spleen-focused US stimulation can lead to substantial changes in the transcriptional profiles of lymphocytes, which are specific to the RA disease state.

In monocytes from RA mice, 9 DEGs with *Q* values <1.88 × 10^−139^ (ranging from *Q* = 2.81 × 10^−289^ to 1.88 × 10^−139^) were identified as compared the US-stimulated RA_US group and non-US-stimulated RA_c group (Fig. 6C). All genes, except *S100a8* and *mt-Co1*, were upregulated upon US stimulation. Similarly, the 9 DEGs (RA_US vs. RA_c) in macrophages of RA mice with *Q* values <6.6 × 10^−35^ (ranging from *Q* = 2.5 × 10^−106^ to 6.6 × 10^−35^), and all but *mt-Nd4* and *Hbb-bs* were downregulated by US stimulation (Fig. 6C). Interestingly, most of the DEGs found in RA mice were not differentially expressed in healthy mice (except *Hbb-bs*, *Jun*, and *Junb*, bolded in Fig. 6), indicating a unique effect of US stimulation on RA mice. Furthermore, half of the DEGs (bold in Fig. 6C) were upregulated in both monocytes and macrophages in RA, demonstrating that US induced overlapping effects in both subgroups of myeloid cells. Regardless of the DEGs, most were upregulated in healthy mice (Fig. S15). These data suggest that US stimulation targeting the spleen can cause substantial changes in the transcriptional profiles of myeloid cells. Of note, these changes are unique to the RA disease state.

To understand the biological significance of transcriptional changes induced by US treatment in splenic T cells, B cells, macrophages, and monocytes, we performed pathway enrichment analysis with DEGs obtained via unsupervised clustering analysis. Among the T cells (Fig. S16), we found enrichment of immune system processes and multiorganism process pathways, which were driven in part by the upregulation of *Junb*, *Klf2*, and *Jund*. *Junb* and *Klf2* play important roles in maintaining the systemic functions of T cells, and their abnormal expression leads to abnormal activation [49] or abnormal migration [50] of T cells. Downregulated genes are involved in inflammatory responses, particularly calprotectin *S100a8* and *S100a9*, which stimulate leukocyte recruitment, induce cytokine secretion, and are biomarkers of inflammation in autoimmune diseases [51]. The upregulation of *KLF2* protein was confirmed by western blot of splenic tissues upon US stimulation (Fig. 6D and E). We also observed upregulation of *S100A9* and no marked changes in *S100A8*. The difference between western blot and scRNA-seq results can be explained by the fact that western blot measures the total protein expression changes in the splenic tissue, whereas scRNA-seq only focuses on specific cell subtypes. In B cells, we found enrichment of pathways, including immune system and inflammatory processes. This can be attributed to the upregulation of the *Fos* gene family, *Fos* and *Fosb*, which encodes leucine zipper proteins that can dimerize with *Jun* family members to generate the activator protein-1 transcription factor complex and promote B cell proliferation, differentiation, and transformation regulators [52]. Western blot was conducted to demonstrate the increase in *c-Jun* protein in splenic tissues in response to US stimulation (Fig. 6D and E). Among monocytes, several pathways, such as immune system processes and multiorganism processes, were enriched, which were driven by the upregulation of Btg2 and H2-D1. BTG2 is a PRMT1-binding protein and the product of a p53-regulated gene and regulates monocyte differentiation [53], whereas H2-D1 belongs to major histocompatibility complex (MHC) class I genes. Downregulated expression of calprotectin *S100A8*, a known inflammation biomarker as mentioned above, and *mt-Co1*, which encodes cytochrome oxidase IV protein, is related to oxidative stress [54]. Another downregulated gene is ribosomal protein *Rpl3*, which is potentially a repressor of inflammatory signaling [55]. In macrophages, we observed downregulation of pathways related to the inflammatory response, driven by the downregulation of calprotectin *S100A8* and *S100A9*. We also found a downregulation of mitochondrial genes *mt-Co1*, *mt-Nd5*, and *mt-Nd4*, which encode cytochrome oxidase IV and nicotinamide adenine dinucleotide: ubiquinone oxidoreductase, respectively, and are associated with oxidative stress [56], which could be another potential mechanism in splenic US stimulation. Overall, pathway enrichment analysis with DEGs identified several potential mechanisms by which US stimulation improves RA, including upregulation of inflammatory signaling regulators, downregulation of inflammatory response, and US-induced oxidative stress in T, B, and myeloid cells.

## Conclusion

House-assembled LFPFU was used to enable spleen-focused US stimulation of a type II collagen-induced arthritis DBA/1J mouse model in this project. It was found that spleen-focused LFPFU therapy exhibited unique clinical efficacy in mice with arthritis. The cell subpopulations among T and myeloid cells were classified using cell-surface markers and multimodel, high-dimensional spleen tissue data. The differences in the number of T and myeloid cell subclusters in the spleens were explained by the unique response of certain cells to spleen-focused US therapy in arthritic mice; in particular, splenic CD8^+^ T cells could serve as an indicator for the effectiveness of splenic-US therapy in treating arthritis. Furthermore, the influence of LFPFU on spleen cells at the transcriptome level was analyzed using scRNA-seq analysis, and the importance of T, B, and myeloid cells in the anti-inflammatory pathways was identified. Overall, this study demonstrates the potential of spleen-focused US stimulation in treating inflammatory diseases. The responsive cell subsets, genes, and associated pathways underlying the reduction in inflammation were mapped, thus providing a comprehensive understanding of the single-cell compendium of splenic immune cells during suborgan US stimulation in arthritic mice.

The present study also maps the first comprehensive immunological landscape of the spleen of RA mice and identifies the changes in the cell subsets, defined by either specific surface marker expression or differential transcriptional expression. These findings potentially provide data that will help in revealing the mechanism of US therapy for autoimmune diseases. Moreover, because the concept of noninvasive treatment is appealing and a larger cohort of clinical studies is needed to unveil the potential of US treatment, the strategy could be applied to the multidimensional profiling of other suborgans and provide a composite methodology to map the specific cell cluster relationships and modulations between different cohorts.

## Materials and Methods

### LFPFU characterization

The operation procedure was modified on the basis of previous literature [57,58]. We used a focused US concave transducer (Panametrics-NDT, Waltham, MA, USA) with a focal length (f) of 33.5 mm and a center frequency of 1 MHz (input voltage was set as 130 mVpp, the spatial peak negative pressure at the focus is 0.35 mPa). In the experiment, the thickness of the skin and the depth of the spleen of the mice were monitored by a real-time palmtop US (Lumify equipped with Liner probe L12-4, Philips N.V.). We used a waveguide filled with degassed water to adjust the distance of the transducer to the spleen to make sure the focus point was located on the spleen. The medical ultrasonic couplant was used between the skin and waveguide. A hydrophone was used in degassed water to measure the normalized sound pressure distribution in the focal plane, as shown in Fig. S4. A 1-s on/5-s off pulsed US signal with a stimulation time of 12 min per day was delivered to mice.

### Mice

Specific pathogen-free grade male DBA/1J mice, weighing between 18 and 22 g, were purchased from the JICUI Laboratory (Jiangsu province, China) at 7 weeks old. Animals were rested at SUN YAT-SEN University’s research animal facility for 2 weeks and were induced with RA at 9 weeks of age. All procedures on mice were carried out according to the protocols approved by the Institutional Animal Care and Use Committee at the Jinan University, second clinical medical campus.

### Induction of RA in mice

RA was induced in DBA/1J mice (SUN YAT-SEN University’s research animal facility) at 9 weeks of age by type II collagen as described [59]. In detail, type II collagen was dissolved in 0.1 mol/l CH_3_COOH with a concentration of 10 mg/ml at 4 °C. The type II collagen solution was then mixed with equal equivalents complete Freund’s adjuvant and fully emulsified to water in an oil state on ice. On day 1, during the initial immunization, 50 μl of emulsified collagen was injected 2 to 3 cm under the skin of the tail of DBA/1J mice; on day 21, emulsified collagen was injected again in the same way. (Five mice were induced and continued observed; on day 28, the mice began to develop joint redness and swelling. The mice were sacrificed, and the knee joints were taken to make paraffin sections and HE-stained to observe arthritic lesions.) In total, 60 DBA/1J mice were induced with RA and grouped into 4 groups randomly: type II collagen-injected mice that received US treatment (RA_US, *n* = 15) or without US treatment (RA_c, *n* = 15); and control non-type II collagen-injected mice with or without US treatment (NT_US, *n* = 15; NT_c, *n* = 15).

### Spleen engagement and arthritis assessment

To allow consistent US treatment, ankle measurements and clinical scoring, the mice were anesthetized with 1% isoflurane during each session and placed on a fixed pad. All animals within an experiment received the same amount of time under anesthetic, regardless of whether they were in US or control cohort. Clinical scores and ankle thickness were also measured while the mice were anesthetized on days 0 to 10 (relative to second collagen injection), and final ankle thicknesses and clinical scores were measured just after euthanasia with 5% isoflurane on day 10. Mice were shaved over their left flank to allow maximum US penetration of the skin on the −1 d of the experiment, and the US-focusing cone filled with degassed water was coupled to the skin with US gel. Control followed the same procedure, but US was not turned on. The daily change in average ankle thickness was measured with a precise caliper (0.01-mm resolution) and clinical scores were determined on the basis of the established method of assessing rodent arthritis index on a 0 to 12 scale [60]. Starting from the second injection of collagen (day 21), the progression of arthritis was scored and recorded every day. Four paws of the mice were visually observed, and the severity of arthritis was documented. The clinical score for each paw ranged from 0 to 3, with 0 indicating no swelling (no inflammation). Clinical score of each paw: 0 = no sign of inflammation; 1 = mild inflammation (metatarsal phalanges joints, individual phalanx, or localized edema); 2 = moderate swelling, but local atrophy on the back or ventral surface of paw; and 3 = highly inflamed swelling in various parts of the paw. The maximum score is 12.

To target the spleen, the real-time spleen depth and dimensions were analyzed from 5 mice and shown in Fig. S2A by Lumify equipped with Liner probe L12-4 (Philips N.V.); after anesthetization of 5 mice, Lumify was used to real-time monitor the location and the size of the spleens. The surface of the spleen is in direct contact with the abdominal wall, so the depth (distance from the skin to the surface of the spleen) was measured by US image (Fig. S2B). The dimensions of the spleen itself were measured through US image and then were confirmed after extraction with a surgical ruler (Fig. S2C).

Body weight was measured daily before anesthetizing the animal by placing the animal in a weighing container on a digital balance weighing instrument (BT25S/BS210S, 220g, 0.1-mg resolution). In order to minimize any circadian effects in which time of day could influence the effectiveness of US treatment, the order of the animals in terms of anesthetization and stimulation was randomly arranged every day. All experiments used the same US parameters applied from day 0 through 10; 1 MHz US at 350 kPa, 1-s on/5-s off bursts with the shallow US-focusing core to target the spleen in animals with collagen injection at day 0.

### Histology (HE), IHC, and IF, and western blot analysis

After the time course of US stimulation for 10 d, the mice’s heart, liver, spleen, lung, kidney and ankle joint tissues were separated (total *n* = 12). These tissues were immediately placed in 4% paraformaldehyde for fixation, and the ankle joint tissue was decalcified with 10% EDTA for 3 weeks, dehydrated, and embedded in paraffin. Other tissues were placed in 10% sucrose, 20% sucrose, and 30% sucrose for dehydration and then embedded in paraffin. Slices (5 μm) were stained with HE. The mice joint sections were scored for changes in synovial proliferation and infiltration of the synovial membrane by mononuclear cells, inflammation, and fibrosis. In addition, mice joint tissues and spleen slices (5 μm) were incubated with primary antibodies against *CD4* (ab245956, Abcam, Cambridge, United Kingdom), *CD8* (ab4055, Abcam), and *CD11C* (GB11059, Servicebio, Gent, Belgium) for 2 h separately, followed by incubation with polymer horseradish peroxidase detection system for 30 min. The final-colored product was developed using the DAB substrate Color Kit (36302ES01, Shanghai Yisheng Biotechnology Company). To perform the IF assay, samples were stained using 4′,6-diamidino-2-phenylindole, sealed with an antifluorescence quenching agent, and observed under the fluorescence microscope after which the images were captured. After sodium dodecyl sulfate polyacrylamide gel electrophoresis separation, proteins of interest were transferred to polyvinylidene difluoride membranes using 110-V constant voltage for 2 h. Membranes were blocked with 5% (w/v) bovine serum albumin (BSA) in TBST buffer (Tris Buffered Saline with Tween-20), and subsequently incubated with primary antibodies *S100A9* (GB111149, Servicebio), *C-Jun* (GB111604, Servicebio), *S100A8* (YN2352, Immuno Way), and *KLF2* (YN3112, Immuno Way) separately and then incubated with secondary antibodies at optimized dilution ratios and incubation time. Western blot results were imaged, and protein band densities were analysed using ImageJ software. The total protein densities in each lane of the SDS-PAGE gel were quantified using loading control for protein normalization.

### Single-cell isolation

Mice were deeply anesthetized using isoflurane (2%) on an online anesthesia machine and real-time monitored. Upon the loss of nociceptive reflexes, spleens were removed and gently homogenized in ice-cold Hank’s Balanced Salt Solution (Life Technologies, 14175-095) on ice. Then, spleens were washed in RPMI 1640 medium (Gibco, C11875500BT) twice, weighted, and taken photos for recording. Then, the spleens were minced and mechanically ground with 70-μm filter strainers, washed by RPMI 1640 medium to obtain single-cell suspensions, centrifugated at 400g × 4 min and discard the supernatants, subjected to 1 ml of ACK RBC lysis buffer (Sigma-Aldrich) for around 1 min, followed by FACS buffer (eBioscience, 00-4222-26) to stop the reaction. The suspension was passed through 70-μm filter cell strainers again if there were flocculent precipitates.

### Mass-tag cell barcoding and antibody staining

Samples of each group were mass-tag cell barcoded. In each sample a unique combination of 5 palladium isotopes (Pd-104, Pd-105, Pd-106, Pd-108, Pd-110) was used to encode 20 unique mass-tag barcodes as described previously [61]. Approximately 1.5 × 10^6^ cells from each group in each sample were barcoded. The cells were washed once with cell staining medium [cell staining medium (CSM), PBS with 0.5% BSA, 0.02% NaN_3_] and then once with PBS for methanol permeabilization. Then, different combinations of palladium-containing mass-tag cell barcoded reagents in dimethyl sulfoxide were added to each sample in 1:100 dimethyl sulfoxide, vortexed, and incubated at 4 °C for 15 min, followed by 3 washes with CSM. The barcoded cells were then resuspended in CSM for antibody staining. Forty-two metal-conjugated antibodies panel were used in the study, as shown in Table S1, including their main manufacturers, clones, and corresponding metal conjugates. Cells were mixed with metal-conjugated surface labeled antibody, generating a final reaction volume of 500 μl and stained on a shaker at room temperature for 30 min. After staining, the cells were washed twice with freshly prepared CSM. Next, the cells were permeabilized with 4% methanol at 4 °C for 10 min. The cells were then washed twice in CSM to remove the remaining methanol. Then, they were stained with 500-μl antibodies on a shaker at room temperature for 30 min. Samples were then washed twice with CSM and incubated overnight at 4 °C with 1 ml of 1:4,000 ^191/193^Ir DNA intercalator (DVS Sciences/fluidigm, Markham, ON) and diluted with 1.6% paraformaldehyde in PBS overnight. On the second day, the cells were washed once with CSM and then washed twice with double-deionized water.

### CyTOF data acquisition and analysis

CyTOF analyses were performed by PLTTech Inc. (Hangzhou, China) according to the previously described protocol [62]. The stained and intercalated samples were washed with PBS before analysis and then resuspended in deionized water containing 20% EQ beads at a concentration of 1 ×10^6^ cells/ml, which contain lanthanum-139, praseodymium-141, terbium-159, thulium-169 and lutetium-175. Stained cells were analyzed on a CyTOF system (Helios) at a rate of 300 times per second. Signal duration (called event length) and iridium intensity were recorded for each event. Single cells were gated on the basis of the fact that single events have lower iridium intensity (because they have less DNA) and lower event length values than aggregates events (Fig. S8). Data were gated to exclude residual normalization beads, fragments, dead cells, and doublets, leaving DNA^+^ CD45^+^ Cisplatin low events for subsequent clustering and high dimensional analyses [63].

### Clustering

All 42 immune cell markers were applied for clustering and visualization. A total of 30,000 cells were selected randomly for visualizing by t-SNE using the R package CyTOF kit [64]. Immune subset cells were defined by the median values of specific expression markers on hierarchical clustering. The comparison between the 2 groups was evaluated by unpaired Student *t* tests. The use of these tests was justified according to the assessment of normality and variance of the data distribution. Differences were considered significant when *P* values are less than 0.05. Data were analyzed using GraphPad Prism (v7).

### scRNA-seq experimental methods, sample preparation, and cell sorting

Eight mice were used for the scRNA-seq experiment, with an *n* = 2 in each of the 4 experiment cohorts. Spleens were dissected from each mouse approximately 4 h post-treatment on the 10th day of stimulation, and samples were kept on ice for the entirety of the experiment. Spleens were processed into single-cell suspensions, and homogenization and staining were performed in 2% BSA in PBS supplemented with 5 mM EDTA.

### scRNA library preparation, sequencing, and statistical analyses

Cell count and viability for samples were determined immediately after single-cell isolations. Cell viability for each sample ranged between 80% and 90%, and cell libraries were made from a target of ~10,000 cells per sample. Libraries were made via the 10× Genomics Chromium Single Cell 3’ Library & Gel Bead Kit (v3) following the manufacturer’s instructions. Libraries were sequenced on an Illumina NovaSeq 6000 sequencing system (paired-end multiplexing run,150 bp) by LC-Bio Technology Co. Ltd., (HangZhou,China). The reading depth of each cell is at least 20,000 times. Sequencing results were demultiplexed using Illumina bcl2fastq software (version2.20) and converted to FASTQ format. Sample demultiplexing, barcode processing, and single-cell 3′ gene counting were conducted using the Cell Ranger pipeline (https://support.10xgenomics.com/single-cell-geneexpression/software/pipelines/latest/what-is-cell-ranger, version 3.1.0), and scRNA-seq data were aligned to the mm10 mouse genome. The Cell Ranger output was loaded into Seurat (version 3.1.1), and the harmony algorithm was employed in the dimensional reduction. On the basis of comparisons of the profiles of the 8 samples, cells were filtered, keeping only those cells whose number of genes detected in each cell is more than 500, and the percentage of mitochondrial genes is less than 0.2. T cells (*Cd3e*, *Cd3d*, *Cd28*, and *Ccl4* as marker genes): clusters 3, 9, 10, 23, 27, 28; B cells (*Ma4a1*, *Cd79a*, and *CD19* as marker genes): clusters 0, 1, 12, 15; NKT cells (*Nkg7* as marker gene):cluster 16; monocytes (*Fcgr3* as marker gene): clusters 2 and 8; DCs (*Siglech*, *Cd83* as marker genes): clusters 19 and 21; granulocytes (*Ly6g*, *Lgals3*, *Itgam*, and *Ly6c2* as marker genes): clusters 6, 11, 14, 22, 25, and 26; macrophages (*C1qa*, *C1qb*, *C1qc*, *Selenop*, *Csf1r*, and *Cd86* as marker genes): cluster 5, 7, 13, and 29; endothelial cells (*Pecam1* and *Cdh5* as marker genes): clusters 17 and 18; erythrocytes (*Gypa* and *Slc4a1* as marker genes): clusters 4 and 20; fibroblasts (*Gdpd2* and *Bgn* as marker genes): cluster 24. Cell trajectory analysis of the myeloid cells including monocytes, macrophages, granulocytes and DCs was performed using the OmicStudio tools (monocle2.0, https://www.omicstudio.cn/analysis/tenXMonocle) according to the literature [43,65]. Cell–cell communication was analyzed using CellPhoneDB (v2.1.1, https://github.com/Teichlab/cellphonedb) with normalized count data as input file according to the literature [44]. The significant ligand–receptor pairs were filtered with a *P* value of less than 0.01. Then, differential expression analysis was performed by using the Wilcox rank sum test on T cells, B cells, monocytes, and macrophages between control and US treatment with either arthritic or healthy mice. Each group of cells was specifically evaluated in this study because of its relevance to the proposed mechanism of action for US stimulation of the anti-inflammatory pathway via the spleen based on previous studies [66–68]. Expression levels of these significantly DEGs are reported in T cells, B cells, monocytes, and macrophages with an adjusted *P* value (*Q* value) using the Bonferroni correction.

## Supplementary Materials

Supplementary Materials Fig. S1. Induction of RA model in DBA/1J mice. Fig. S2. Real-time monitor of spleen in DBA/1J mice by Lumify. Fig. S3. Time course of arthritis in none US treated mice. Fig. S4. Schematic diagram of low-frequency focused US stimulation of mouse spleen. Fig. S5. Body weight correlated with the duration of US therapy. Fig. S6. HE staining images of suborgans of mice after receiving spleen-focused US or no US stimulation in 4 cohorts. Fig. S7. The heterogeneity of the local immune environment in splenic cells from RA-model mice. Fig. S8. Gating strategy of splenic lymphoid cells (CD3^+^ CD19-CD11b-Ly6G-F4_80-). Fig. S9. Gating strategy of splenic myeloid cells (CD3^+^ CD19-CD11b-Ly6G-F4_80-). Fig. S10. Heatmaps showing the differential expression of 42 markers among 26 cell clusters in T cells (a) and myeloid cells (b). Fig. S11. Correlations between the results of the CyTOF mass spectrometry and scRNA-seq analysis of cell types. Fig. S12. Pseudotime trajectory analysis of all 28, 657 myeloid cells generated using Monocle. Fig. S13. Heatmaps showing the number of interactions between cell types in the spleens from (a) NT_c and (b) NT_US groups. Fig. S14. Bubble plots showing the special ligand–receptor pairs between fibroblasts with endothelial cells (a), fibroblasts with macrophages (b) and fibroblasts with monocytes (c) in 4 groups. Fig. S15. The dot plot shows the DEGs in B cells, T cells, monocytes and macrophages from healthy mice relative to controls. Fig. S16. GO biological processes upregulated in splenic T cells, B cells, macrophages and monocytes following US stimulation in RA mice. Table S1. A summary of antibodies used for the panel. Table S2. DEGs in B, T, monocytes and macrophages between US and no US stimulation in both arthritic and healthy mice.
