# Supplementary material for: Noninvasive Low-Frequency Pulsed Focused Ultrasound Therapy for Rheumatoid Arthritis in Mice
Source: Research (Wash D C). 2022 Dec 19;2022:0013. doi: 10.34133/research.0013 (PMC11407525; doi:10.34133/research.0013)
Supplement: Supplementary Materials — Fig. S1. Induction of RA model in DBA/1J mice. Fig. S2. Real-time monitor of spleen in DBA/1J mice by Lumify. Fig. S3. Time course of arthritis in none US treated mice. Fig. S4. Schematic diagram of low-frequency focused US stimulation of mouse spleen. Fig. S5. Body weight correlated with the duration of US therapy. Fig. S6. HE staining images of suborgans of mice after receiving spleen-focused US or no US stimulation in 4 cohorts. Fig. S7. The heterogeneity of the local immune environment in splenic cells from RA-model mice. Fig. S8. Gating strategy of splenic lymphoid cells (CD3+ CD19-CD11b-Ly6G-F4_80-). Fig. S9. Gating strategy of splenic myeloid cells (CD3+ CD19-CD11b-Ly6G-F4_80-). Fig. S10. Heatmaps showing the differential expression of 42 markers among 26 cell clusters in T cells (a) and myeloid cells (b). Fig. S11. Correlations between the results of the CyTOF mass spectrometry and scRNA-seq analysis of cell types. Fig. S12. Pseudotime trajectory analysis of all 28, 657 myeloid cells generated using Monocle. Fig. S13. Heatmaps showing the number of interactions between cell types in the spleens from (a) NT_c and (b) NT_US groups. Fig. S14. Bubble plots showing the special ligand–receptor pairs between fibroblasts with endothelial cells (a), fibroblasts with macrophages (b) and fibroblasts with monocytes (c) in 4 groups. Fig. S15. The dot plot shows the DEGs in B cells, T cells, monocytes and macrophages from healthy mice relative to controls. Fig. S16. GO biological processes upregulated in splenic T cells, B cells, macrophages and monocytes following US stimulation in RA mice. Table S1. A summary of antibodies used for the panel. Table S2. DEGs in B, T, monocytes and macrophages between US and no US stimulation in both arthritic and healthy mice. [file research.0013.f1.docx]

Supplementary Materials

**Supplementary Figures**

**
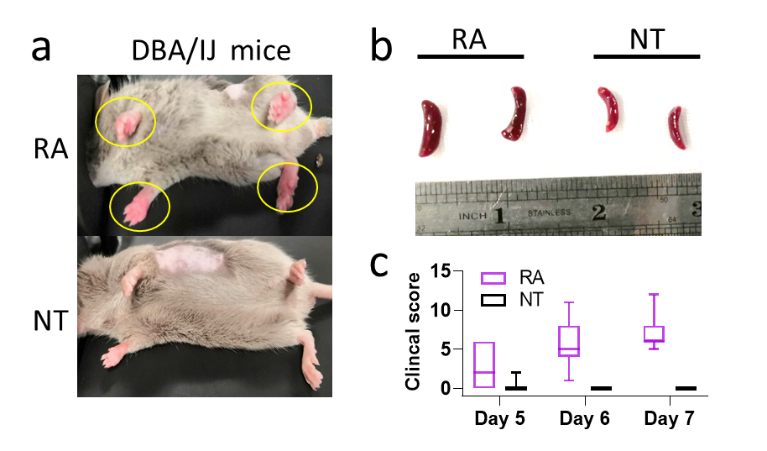
**

**Fig. S1. Induction of rheumatoid arthritis model in DBA/1J mice.** **a** Graph of paw swelling degree after (RA) and before (NT) modeling of DBA/1J mice. **b** Comparison of spleen tissue size of DBA/1J mice after (RA) and before (NT) modeling. **c** Clinical score of paw swelling degree of mice after the second injection of collagen II.


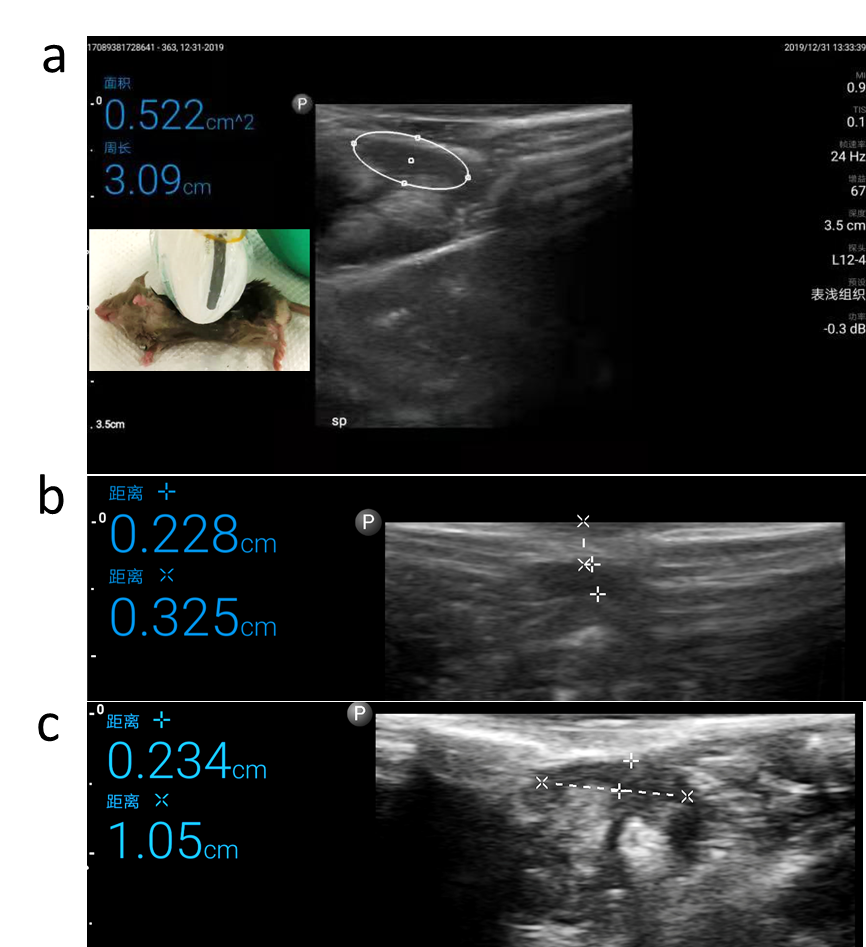


**Fig. S2. Real-time monitor of spleen in DBA/1J mice by Lumify. a** The area and perimeter of the spleen under real-time ultrasound image collected by Lumify equipped with Liner probe L12-4 (Philips N.V.). (insert: the handheld ultrasound coupled to the DBA/1J mice with medical ultrasonic couplant.) **b** The subcutaneous depth and longitudinal section thickness of the spleen under real-time ultrasound image collected by Lumify equipped with Liner probe L12-4 (Philips N.V.). **c** The transverse section length of the spleen under real-time ultrasound image.


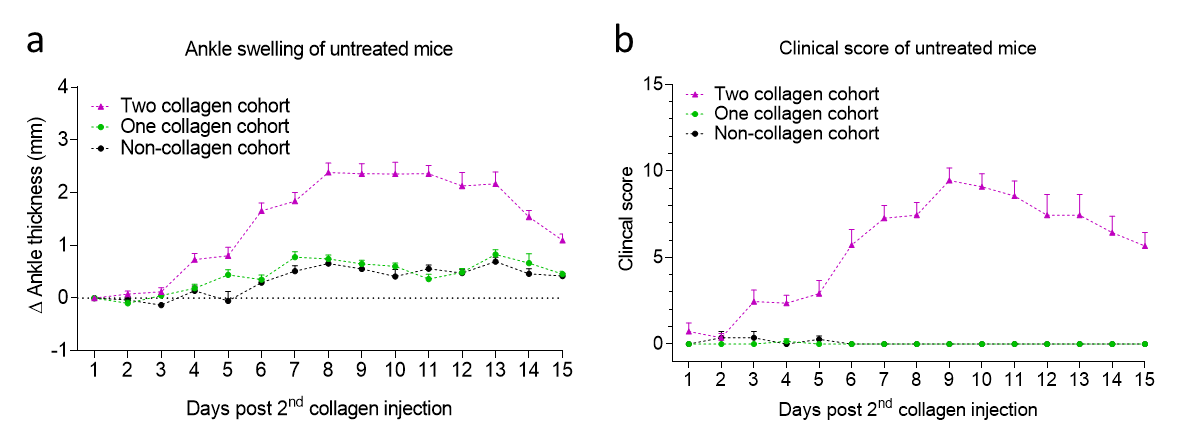


**Fig. S3.** **Time course of arthritis in none US treated mice.** Changes in ankle swelling (**a)** and clinical score **(b)** of three cohorts of mice. The first arthritic cohort was injected on day -28 (first injection) and again on day 0 (second injection) with type II collagen. The second cohort only received collagen injection on day -28. The third cohort did not receive collagen injection. n = 10 for each cohort.


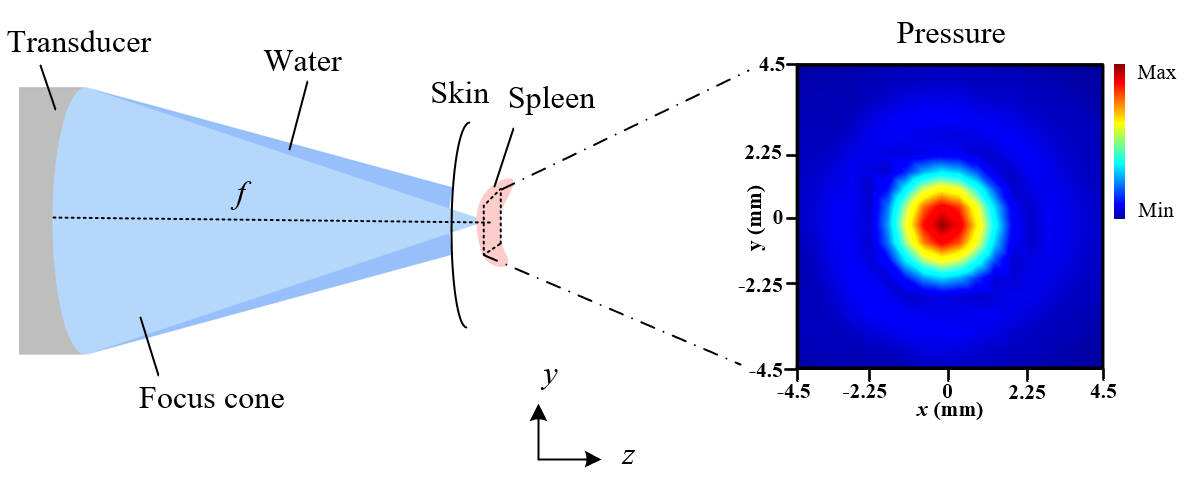


**Fig. S4.** **Schematic diagram of low-frequency focused ultrasound stimulation of mouse spleen.** *f* represents the focal length, the blue area is the focus cone and filled with degassed water, and the cone tip is located in the spleen. The picture on the right shows the normalized sound pressure distribution in the x-y plane at the focal point measured by a hydrophone in degassed water.

**Fig. S5.** **Body weight correlated with the duration of US therapy.** The measured daily values for the bodyweight of mice in four cohorts from day 1 to day 10 are shown. Total n = 60.


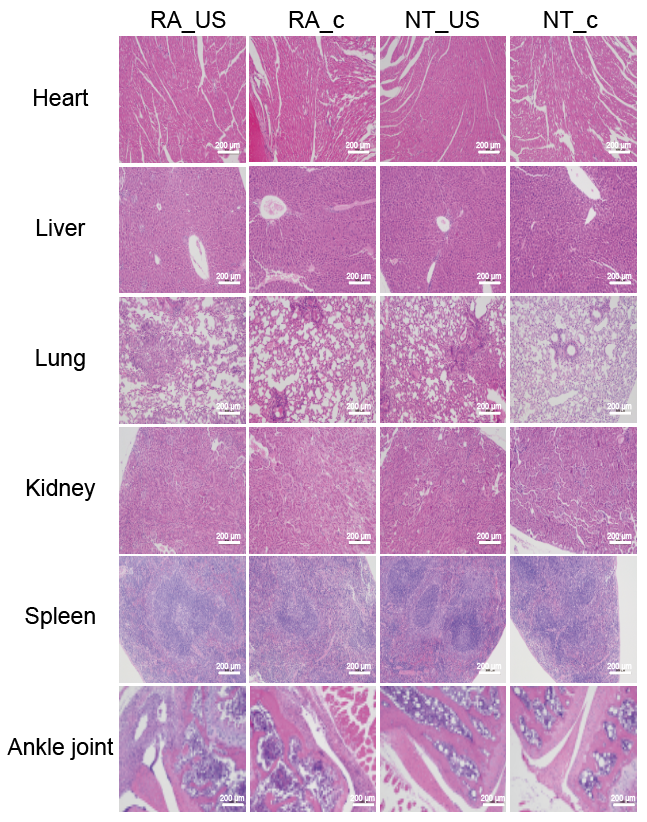


**Fig. S6.** **HE staining images of sub-organs of mice after receiving spleen-focused US or no US stimulation in four cohorts.** After receiving spleen-focused US stimulation, the hearts, livers, spleens, lungs, and kidneys of mice were separated, and performed HE staining. Red represents the cytoplasm; blue represents the nucleus. Scale bar: 200 μm.


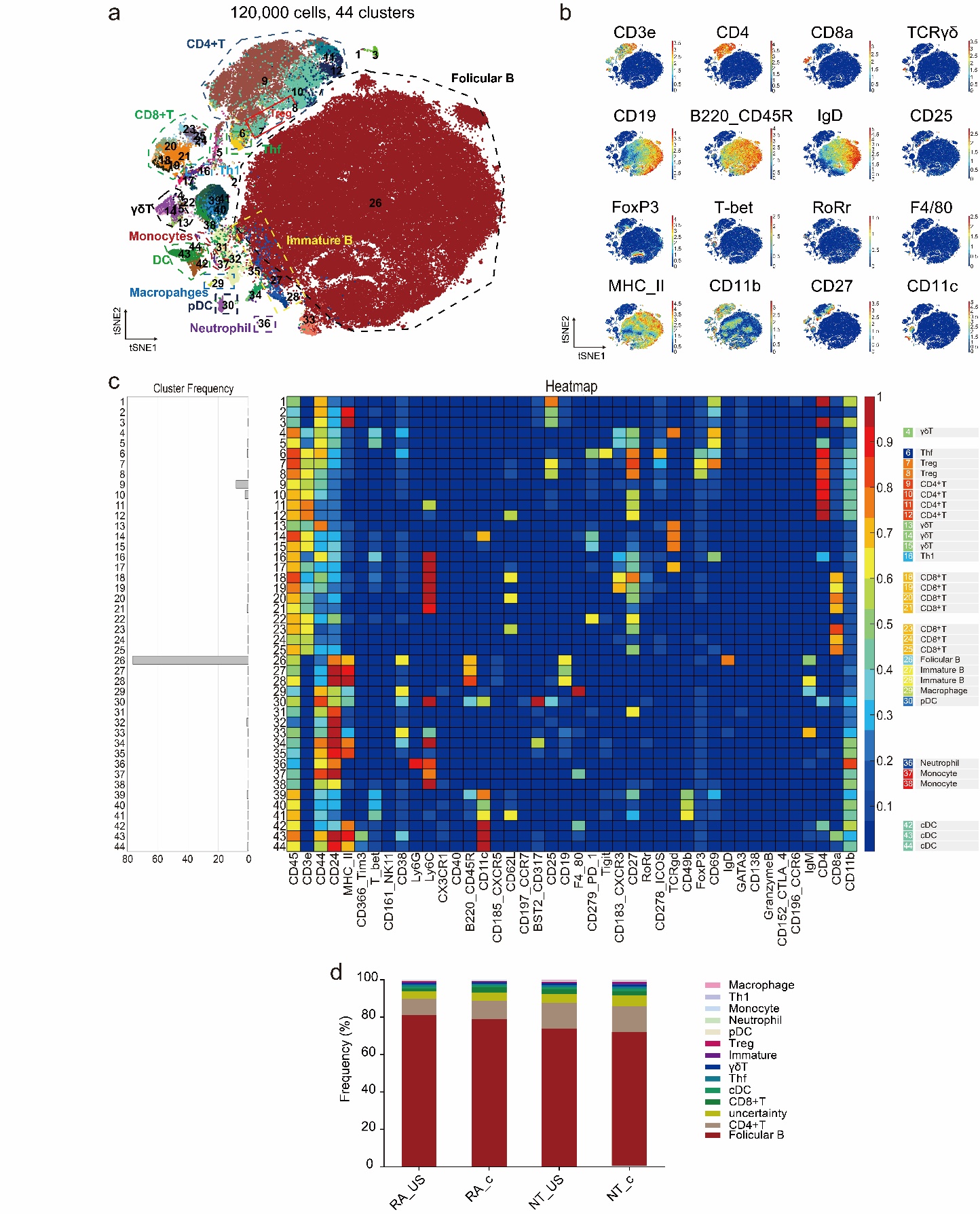


**Fig. S7.** **The heterogeneity of the local immune environment in splenic cells from RA model mice.** **a** t-SNE plot of 44 identified clusters **b** color-coded according to the expression of 16 marker genes for T cell subtypes. **c** Heatmap showing the differential expression of 42 markers among the 44 clusters. Each cluster was identified as a known cell type according to cell-surface marker expression. **d** Frequencies of the 14 cell subsets in four mice groups.

**
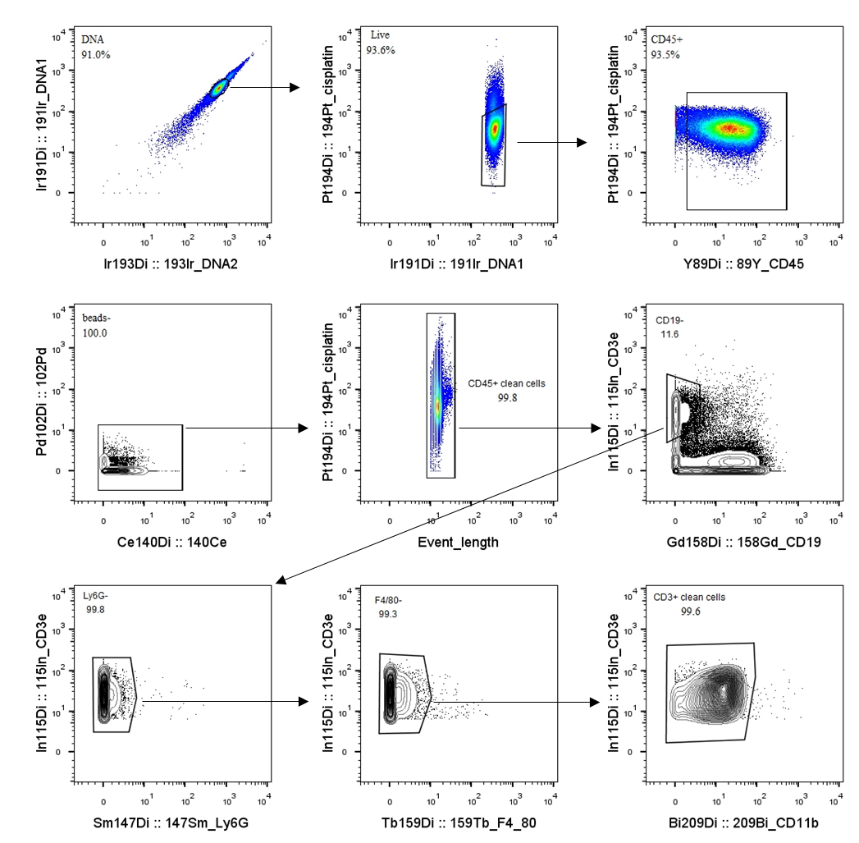
**

**Fig. S8.** Gating strategy of splenic lymphoid cells (CD3^+^ CD19-CD11b-Ly6G-F4_80-).

**
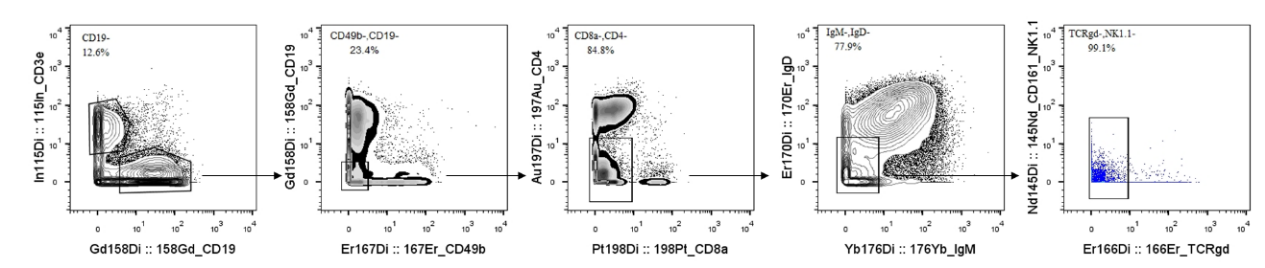
**

**Fig. S9.** Gating strategy of splenic myeloid cells (CD3^+^ CD19-CD11b-Ly6G-F4_80-).


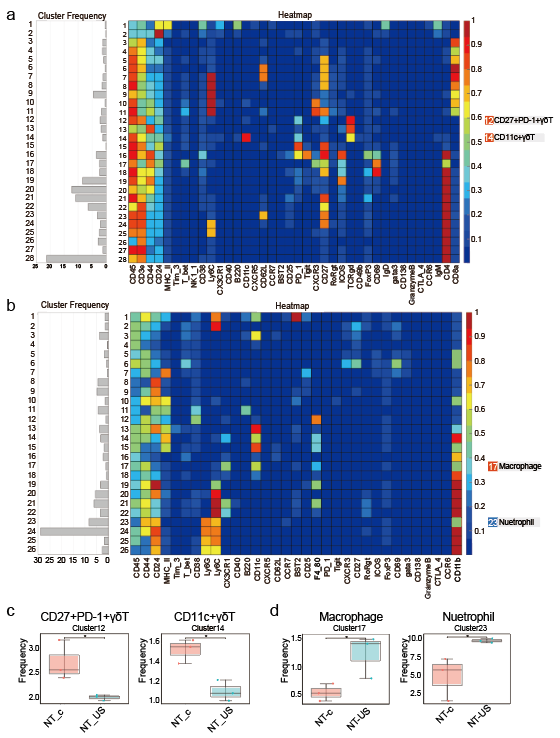


**Fig. S10.** **Heatmaps showing the differential expression of 42 markers among 26 cell clusters in T cells** (**a**) **and myeloid cells** (**b**). Certain clusters were identified as known cell types according to typically expressed markers. Frequencies of two sub-clusters in the splenic T cells (**c**) and myeloid cells (**d**) between the NT_c and NT_US groups. *p<0.05; **p<0.01; ***p<0.001.


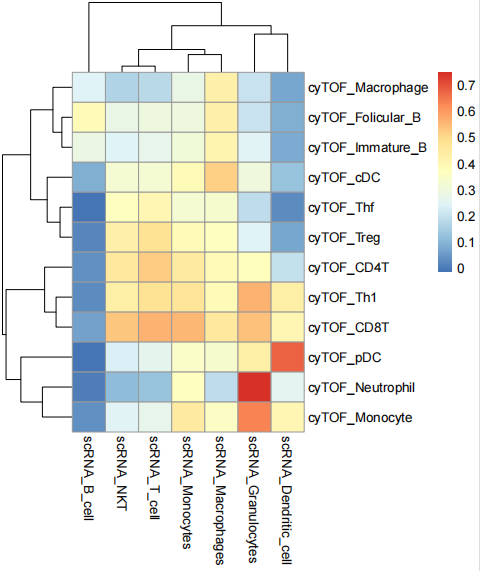


**Fig. S11.** **Correlations between the results of the cytometry time-of-flight (CyTOF) mass spectrometry and single-cell RNA-sequencing (scRNA-seq) analysis of cell types.**


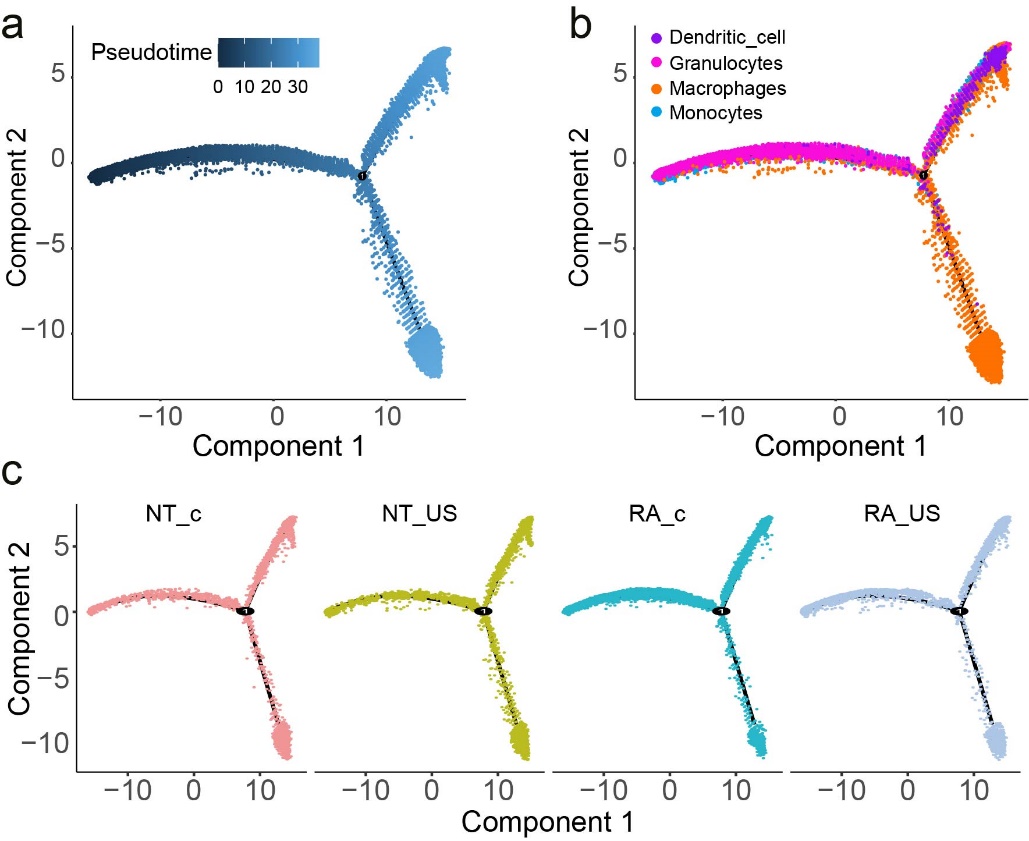


**Fig. S12.** **Pseudotime trajectory analysis of all 28, 657 myeloid cells generated using Monocle.** **a** Pseudotime trajectory of all myeloid cells. **b** Pseudotime trajectory based on cell type coloring. **c** Pseudotime trajectory of myeloid cells based on group coloring. Each point in the diagram represents a cell, and cells with similar states are clustered together. Each branch point represents a possible decision point in the cell biological process.


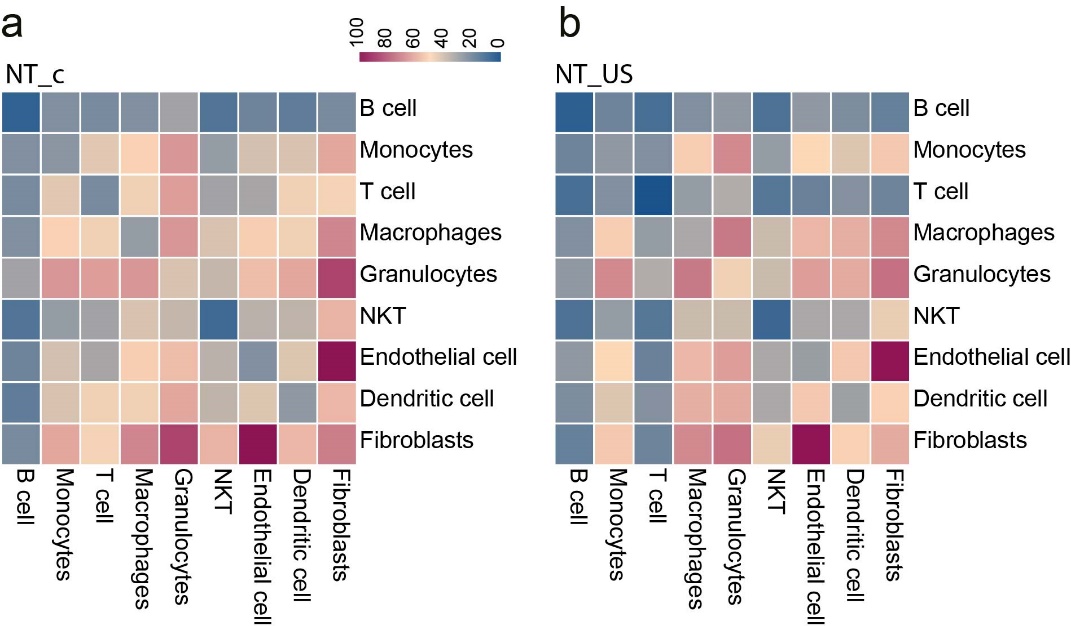


**Fig. S13.** **Heatmaps showing the number of interactions between cell types in the spleens from** (**a**) **NT_c and** (**b**) **NT_US groups**. Rose red and blue represent high and low number of interactions, respectively.


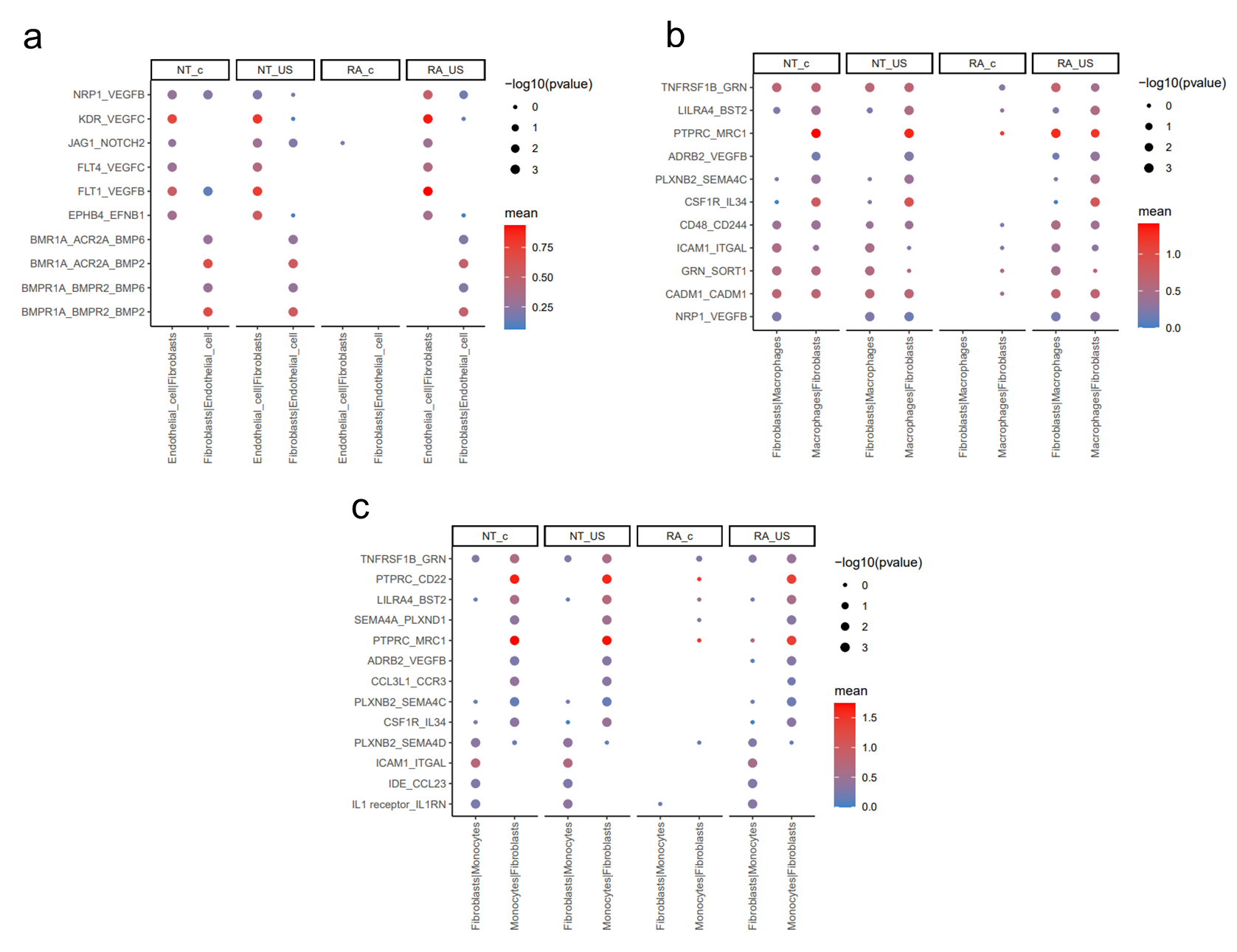


**Fig. S14.** Bubble plots showing the special ligand-receptor pairs between fibroblasts with endothelial cells (a), fibroblasts with macrophages (b) and fibroblasts with monocytes (c) in four groups. P-value was set as <0.05.


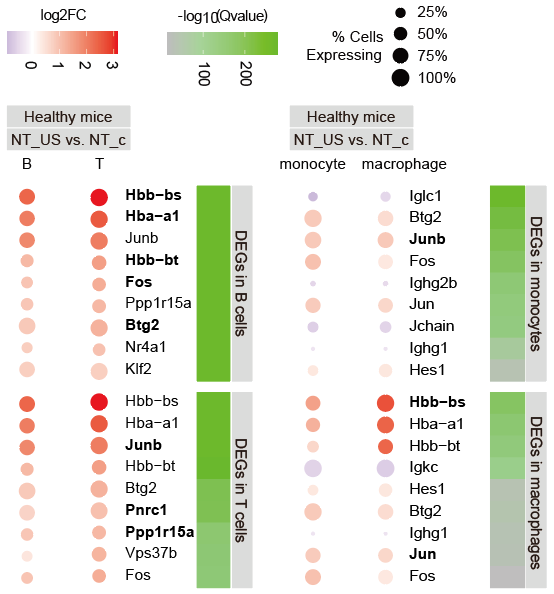


**Fig. S15.** **The dot plot shows the DEGs in B cells, T cells, monocytes and macrophages from healthy mice relative to controls.** The size of each circle represents the percentage of cells within each cell type that expresses the gene listed along with the color of each circle, which represents log_2_ fold change of average scaled expression of NT_US relative to the NT_c group. -log_10_ (Q value) for each gene in all the cells are shown in shades of green. All DEGs listed had a Q value < 9.98e-23 according to Benjamini-Hochberg tests.


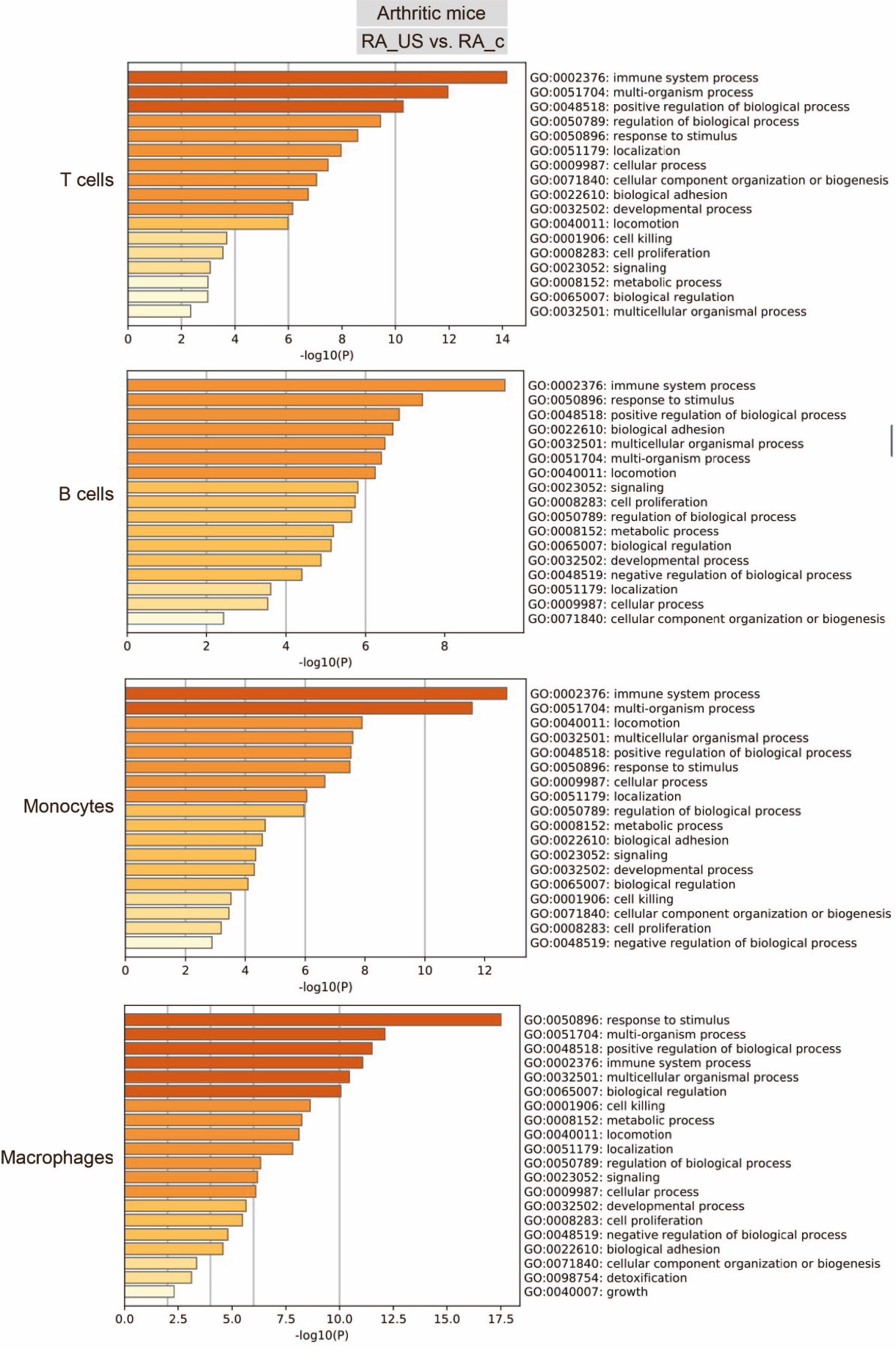


**Fig. S16.** **GO biological processes upregulated in splenic T cells, B cells, macrophages and monocytes following US stimulation in RA mice.** *p* values were derived from Fisher's exact test.

**Supplementary Tables**

**Table S1.** A summary of antibodies used for the panel.

| Metals conjugated | Antibodies | Clone | Source | Identifiers |
| --- | --- | --- | --- | --- |
| 89Y | CD45 | 30-F11 | BioLegend | 103102 |
| 115In | CD3e | 145-2C11 | BioLegend | 100302 |
| 139La | CD44 | IM7 | BioLegend | 103002 |
| 141Pr | CD24 | M1/69 | BioLegend | 101802 |
| 142Nd | MHC II | Y3P | Bio X Cell | BE0178 |
| 143Nd | TIM-3 | RMT 3-23 | BioLegend | 119702 |
| 144Nd | T-bet | 4B10 | BioLegend | 644802 |
| 145Nd | NK1.1 | PK136 | BioLegend | 108702 |
| 146Nd | CD38 | 90 | BioLegend | 102702 |
| 147Sm | Ly6G | IA8 | BioLegend | 127602 |
| 148Nd | Ly6C | HK1.4 | BioLegend | 128002 |
| 149Sm | CX3CR1 | SA011F11 | BioLegend | 149002 |
| 150Nd | CD40 | 3/23 | BioLegend | 124601 |
| 151Eu | B220 | RA3-6B2 | BioLegend | 103202 |
| 152Sm | CD11c | N418 | BioLegend | 117302 |
| 153Eu | CXCR5 | L138D7 | BioLegend | 145502 |
| 154Sm | CD62L | MEL-14 | BioLegend | 104402 |
| 155Gd | CCR7 | 4B12 | BioLegend | 120101 |
| 156Gd | BST2 | 44E9R | R&D Systems | MAB8660 |
| 157Gd | CD25 | 3C7 | BioLegend | 101902 |
| 158Gd | CD19 | 6D5 | BioLegend | 115502 |
| 159Tb | F4/80 | C1:A3-1 | Bio-Rad | MCA497G |
| 160Gd | CD279(PD-1) | 29F.1A12 | BioLegend | 135202 |
| 161Dy | Tigit | 2190A | R&D Systems | MAB72671 |
| 162Dy | CXCR3 | CXCR3-173 | BioLegend | 126502 |
| 163Dy | CD27 | LG.3A10 | BioLegend | 124202 |
| 164Dy | RoRγt | 600214 | R&D Systems | MAB6109 |
| 165Ho | ICOS | C398.4A | BioLegend | 313502 |
| 166Er | TCRgd | GL3 | BioLegend | 118101 |
| 167Er | CD49b | DX5 | BioLegend | 108902 |
| 168Er | FoxP3 | FJK-16S | eBioscience | 14-5773-82 |
| 169Tm | CD69 | H1.2F3 | BioLegend | 104502 |
| 170Er | IgD | 11-26C.2a | BioLegend | 405702 |
| 171Yb | gata3 | TWAJ | eBioscience | 14-9966-82 |
| 172Yb | CD138 | 281-2 | BioLegend | 142502 |
| 173Yb | GranzymeB | GB11 | BioLegend | 372202 |
| 174Yb | CTLA-4 | UC10-4B9 | BioLegend | 106302 |
| 175Lu | CCR6 | 29-2L17 | BioLegend | 129802 |
| 176Yb | IgM | RMM-1 | BioLegend | 406502 |
| 197Au | CD4 | RM4-5 | BioLegend | 100520 |
| 198Pt | CD8a | 53-6.7 | BioLegend | 100716 |
| 209Bi | CD11b | M1/70 | BioLegend | 101202 |
| 89Y | CD45 | 30-F11 | BioLegend | 103102 |
| 115In | CD3e | 145-2C11 | BioLegend | 100302 |

**Table S2.** Differentially expressed genes in B, T, monocytes and macrophages between US and noUS stimulation in both arthritic and healthy mice

| B Cell Response | | | | | | | | | | |
| --- | --- | --- | --- | --- | --- | --- | --- | --- | --- | --- |
| RA_US vs. RA_c, DEGs | | | | | | | | | | |
| Gene Name | | log2FC | | P vlaue | | Q value | | pct.1 | | pct.2 |
| *Fos* | | 1.68 | | 0.00E+00 | | 0.00E+00 | | 0.48 | | 0.15 |
| *Hbb-bs* | | 1.36 | | 0.00E+00 | | 0.00E+00 | | 0.89 | | 0.58 |
| *Fosb* | | 1.15 | | 0.00E+00 | | 0.00E+00 | | 0.40 | | 0.12 |
| *Dusp1* | | 1.05 | | 0.00E+00 | | 0.00E+00 | | 0.40 | | 0.11 |
| *Hba-a1* | | 1.03 | | 0.00E+00 | | 0.00E+00 | | 0.77 | | 0.39 |
| *Btg2* | | 0.81 | | 0.00E+00 | | 0.00E+00 | | 0.72 | | 0.53 |
| *Ier2* | | 0.75 | | 0.00E+00 | | 0.00E+00 | | 0.38 | | 0.15 |
| *Hbb-bt* | | 0.71 | | 0.00E+00 | | 0.00E+00 | | 0.40 | | 0.12 |
| *Camp* | | -0.53 | | 0.00E+00 | | 0.00E+00 | | 0.51 | | 0.93 |
| NT_US vs. NT_c, DEGs | | | | | | | | | | |
| Gene Name | | log2FC | | P vlaue | | Q value | | pct.1 | | pct.2 |
| *Hbb-bs* | | 2.39 | | 0.00E+00 | | 0.00E+00 | | 0.66 | | 0.12 |
| *Hba-a1* | | 2.03 | | 0.00E+00 | | 0.00E+00 | | 0.62 | | 0.08 |
| *Junb* | | 1.89 | | 0.00E+00 | | 0.00E+00 | | 0.63 | | 0.35 |
| *Hbb-bt* | | 1.11 | | 0.00E+00 | | 0.00E+00 | | 0.39 | | 0.02 |
| *Fos* | | 0.93 | | 0.00E+00 | | 0.00E+00 | | 0.29 | | 0.10 |
| *Ppp1r15a* | | 0.90 | | 0.00E+00 | | 0.00E+00 | | 0.35 | | 0.14 |
| *Btg2* | | 0.85 | | 0.00E+00 | | 0.00E+00 | | 0.76 | | 0.64 |
| *Nr4a1* | | 0.79 | | 0.00E+00 | | 0.00E+00 | | 0.24 | | 0.04 |
| *Klf2* | | 0.77 | | 0.00E+00 | | 0.00E+00 | | 0.64 | | 0.41 |
| T Cell Response | | | | | | | | | | |
| RA_US vs. RA_c, DEGs | | | | | | | | | | |
| Gene Name | | log2FC | | P vlaue | | Q value | | pct.1 | | pct.2 |
| *S100a8* | | -1.24 | | 1.00E-305 | | 3.00E-301 | | 0.62 | | 1.00 |
| *S100a9* | | -1.06 | | 1.91E-283 | | 6.81E-279 | | 0.82 | | 1.00 |
| *Junb* | | 2.04 | | 1.33E-272 | | 4.74E-268 | | 0.83 | | 0.34 |
| *Ngp* | | -0.88 | | 4.86E-139 | | 1.74E-134 | | 0.30 | | 0.78 |
| *Klf2* | | 1.12 | | 2.36E-114 | | 8.43E-110 | | 0.78 | | 0.46 |
| *Ubc* | | 0.89 | | 2.49E-112 | | 8.90E-108 | | 0.77 | | 0.45 |
| *Pnrc1* | | 0.87 | | 1.25E-105 | | 4.47E-101 | | 0.77 | | 0.43 |
| *Ppp1r15a* | | 1.02 | | 9.73E-101 | | 3.48E-96 | | 0.44 | | 0.08 |
| *Jund* | | 0.84 | | 4.82E-97 | | 1.72E-92 | | 0.85 | | 0.61 |
| NT_US vs. NT_c, DEGs | | | | | | | | | | |
| Gene Name | | log2FC | | P vlaue | | Q value | | pct.1 | | pct.2 |
| *Hbb-bs* | | 3.14 | | 0.00E+00 | | 0.00E+00 | | 0.85 | | 0.08 |
| *Hba-a1* | | 2.56 | | 0.00E+00 | | 0.00E+00 | | 0.83 | | 0.07 |
| *Junb* | | 2.03 | | 0.00E+00 | | 0.00E+00 | | 0.84 | | 0.46 |
| *Hbb-bt* | | 1.52 | | 2.38E-284 | | 8.49E-280 | | 0.53 | | 0.01 |
| *Btg2* | | 1.23 | | 1.58E-224 | | 5.66E-220 | | 0.82 | | 0.58 |
| *Pnrc1* | | 1.00 | | 8.62E-222 | | 3.08E-217 | | 0.79 | | 0.51 |
| *Ppp1r15a* | | 1.14 | | 3.76E-171 | | 1.34E-166 | | 0.46 | | 0.10 |
| *Vps37b* | | 1.18 | | 6.41E-165 | | 2.29E-160 | | 0.55 | | 0.19 |
| *Fos* | | 1.31 | | 3.41E-160 | | 1.22E-155 | | 0.44 | | 0.09 |
| Monocyte Cell Response | | | | | | | | | | |
| RA_US vs. RA_c, DEGs | | | | | | | | | | |
| Gene Name | log2FC | | P vlaue | | Q value | | pct.1 | | pct.2 | |
| *Btg2* | 0.79 | | 7.88E-294 | | 2.81E-289 | | 0.75 | | 0.62 | |
| *H2-D1* | 0.35 | | 7.13E-243 | | 2.55E-238 | | 1.00 | | 0.99 | |
| *Hbb-bs* | 0.47 | | 2.48E-204 | | 8.86E-200 | | 0.85 | | 0.61 | |
| *S100a8* | -0.58 | | 1.24E-195 | | 4.43E-191 | | 0.92 | | 1.00 | |
| *Jun* | 0.70 | | 2.38E-167 | | 8.51E-163 | | 0.60 | | 0.46 | |
| *mt-Co1* | -0.42 | | 8.15E-160 | | 2.91E-155 | | 0.94 | | 0.97 | |
| *Ier5* | 0.55 | | 6.79E-153 | | 2.43E-148 | | 0.60 | | 0.40 | |
| *Oaz1* | 0.27 | | 2.37E-149 | | 8.45E-145 | | 0.95 | | 0.90 | |
| *Rpl3* | 0.28 | | 5.27E-144 | | 1.88E-139 | | 0.95 | | 0.93 | |
| NT_US vs. NT_c, DEGs | | | | | | | | | | |
| Gene Name | log2FC | | P vlaue | | Q value | | pct.1 | | pct.2 | |
| *Iglc1* | -0.93 | | 0.00E+00 | | 0.00E+00 | | 0.15 | | 0.57 | |
| *Btg2* | 0.84 | | 1.03E-249 | | 3.69E-245 | | 0.84 | | 0.77 | |
| *Junb* | 0.85 | | 4.56E-227 | | 1.63E-222 | | 0.75 | | 0.70 | |
| *Fos* | 0.98 | | 4.71E-199 | | 1.68E-194 | | 0.67 | | 0.51 | |
| *Ighg2b* | -0.54 | | 3.85E-163 | | 1.38E-158 | | 0.02 | | 0.21 | |
| *Jun* | 0.82 | | 3.92E-147 | | 1.40E-142 | | 0.60 | | 0.50 | |
| *Jchain* | -0.62 | | 1.12E-139 | | 4.00E-135 | | 0.23 | | 0.51 | |
| *Ighg1* | -0.33 | | 1.82E-81 | | 6.52E-77 | | 0.01 | | 0.11 | |
| *Hes1* | 0.38 | | 1.45E-40 | | 5.17E-36 | | 0.22 | | 0.15 | |
| Macrophage Cell Response | | | | | | | | | | |
| RA_US vs. RA_c, DEGs | | | | | | | | | | |
| Gene Name | log2FC | | P vlaue | | Q value | | pct.1 | | pct.2 | |
| *S100a8* | -1.13 | | 7.09E-111 | | 2.53E-106 | | 0.79 | | 1.00 | |
| *Camp* | -0.98 | | 6.85E-106 | | 2.45E-101 | | 0.44 | | 0.93 | |
| *S100a9* | -0.95 | | 2.74E-98 | | 9.80E-94 | | 0.92 | | 1 | |
| *mt-Co1* | -0.76 | | 4.55E-86 | | 1.62E-81 | | 0.92 | | 0.97 | |
| *mt-Nd5* | -0.70 | | 1.92E-71 | | 6.87E-67 | | 0.86 | | 0.93 | |
| *mt-Nd4* | -0.52 | | 3.36E-46 | | 1.20E-41 | | 0.98 | | 0.99 | |
| *Hbb-bs* | 1.28 | | 2.31E-44 | | 8.25E-40 | | 0.99 | | 0.89 | |
| *Lcn2* | -0.86 | | 1.85E-39 | | 6.61E-35 | | 0.27 | | 0.58 | |
| *Junb* | 0.78 | | 2.56E-33 | | 9.13E-29 | | 0.73 | | 0.51 | |
| NT_US vs. NT_c, DEGs | | | | | | | | | | |
| Gene Name | log2FC | | P vlaue | | Q value | | pct.1 | | pct.2 | |
| *Hbb-bs* | 2.66 | | 8.17E-197 | | 2.92E-192 | | 0.84 | | 0.53 | |
| *Hba-a1* | 2.41 | | 1.38E-169 | | 4.93E-165 | | 0.80 | | 0.48 | |
| *Hbb-bt* | 2.39 | | 4.47E-150 | | 1.60E-145 | | 0.57 | | 0.19 | |
| *Igkc* | -0.66 | | 6.25E-118 | | 2.23E-113 | | 0.90 | | 0.99 | |
| *Hes1* | 0.75 | | 5.78E-54 | | 2.07E-49 | | 0.43 | | 0.30 | |
| *Btg2* | 0.56 | | 2.97E-49 | | 1.06E-44 | | 0.63 | | 0.63 | |
| *Ighg1* | -0.36 | | 5.73E-41 | | 2.05E-36 | | 0.01 | | 0.13 | |
| *Jun* | 0.64 | | 3.64E-37 | | 1.30E-32 | | 0.55 | | 0.48 | |
| *Fos* | 0.36 | | 3.81E-20 | | 1.36E-15 | | 0.55 | | 0.56 | |

**References**

1. J.A. Sparks, Rheumatoid arthritis, Annals of Internal Medicine, vol. 170, pp. ITC1-ITC16, 2019.
2. Y. Jamilloux, T. El Jammal, L. Vuitton et al., "JAK inhibitors for the treatment of autoimmune and inflammatory diseases," Autoimmunity Reviews, vol. 18, no. 11, pp. 102390, 2019.
3. Z. Chen, A. Bozec, A. Ramming, G. Schett, "Anti-inflammatory and immune-regulatory cytokines in rheumatoid arthritis," Nature Reviews Rheumatology, vol. 15, no. 1, pp. 9-17, 2019.
4. F.A. Koopman, S.S. Chavan, S. Miljko, S. Grazio, S. Sokolovic, P.R. Schuurman, A.D. Mehta, Y.A. Levine, M. Faltys, R. Zitnik, K.J. Tracey, P.P. Tak, “Vagus nerve stimulation inhibits cytokine production and attenuates disease severity in rheumatoid arthritis, ” Proceedings of the National Academy of Sciences of the United States of America, vol. 113, pp.8284-8289, 2016.
5. K.J. Tracey, “The inflammatory reflex”, Nature, vol.420 pp. 853-859, 2002.
6. K.J. Tracey, “Reflex control of immunity”, Nature Reviews Immunology, vol.9, pp.418-428, 2009.
7. K.J. Tracey, “Reflexes in Immunity”, Cell, vol,164, pp,343-344, 2016.
8. V.A. Pavlov, K.J. Tracey, “The vagus nerve and the inflammatory reflex--linking immunity and metabolism”, Nature Reviews Endocrinology, vol.8 pp.743-754,2012.
9. T.S. Huerta, A. Devarajan, T. Tsaava, et al., "Targeted peripheral focused ultrasound stimulation attenuates obesity-induced metabolic and inflammatory dysfunctions," Scientific Reports, vol. 11, no. 1, pp. 5083, 2021.
10. D.P. Zachs, S.J. Offutt, R.S. Graham et al., "Noninvasive ultrasound stimulation of the spleen to treat inflammatory arthritis," Nature Communication, vol. 10, no. 1, pp. 951, 2019.
11. V. Cotero, Y. Fan, T. Tsaava et al., "Noninvasive sub-organ ultrasound stimulation for targeted neuromodulation," Nature Communication, vol. 10, no. 1, pp. 952, 2019.
12. N. Choudhary, L.K. Bhatt, K.S. Prabhavalkar, "Experimental animal models for rheumatoid arthritis," Immunopharmacol Immunotoxicol, vol. 40, no. 3, pp. 193-200, 2018.
13. D.D. Brand, K.A. Latham, E.F. Rosloniec, “Collagen-induced arthritis”, Nature Protocols, wol.2 ,pp. 1269-1275, 2017.
14. B. Ajami, N. Samusik, P. Wieghofer et al., "Single-cell mass cytometry reveals distinct populations of brain myeloid cells in mouse neuroinflammation and neurodegeneration models", Nature Neuroscience, vol. 21, no. 4, pp. 541-551, 2018.
15. F.C. Grandi, R. Baskar, P. Smeriglio et al., "Single-cell mass cytometry reveals cross-talk between inflammation-dampening and inflammation-amplifying cells in osteoarthritic cartilage", Science Advances, vol. 6, no. 11, pp. eaay5352, 2020. 6 (2020).
16. D.M. Lee, D.S. Friend, M.F. Gurish et al., "Mast cells: A cellular link between autoantibodies and inflammatory arthritis," Science, vol. 597, no. 5587, pp. 1689-1692, 2002.
17. B.A. Binstadt, J.L. Hebert, A. Ortiz-Lopez et al., "The same systemic autoimmune disease provokes arthritis and endocarditis via distinct mechanisms," Proceedings of the National Academy of Sciences of the United States of America, vol. 106, no. 39, pp. 16758-16763, 2009.
18. A.E. Cardona, D. Huang, M.E. Sasse et al., "Isolation of murine microglial cells for RNA analysis or flow cytometry," Nature Protocols, vol. 1, no. 4, pp. 1947-1951, 2006.
19. N. Samusik, Z. Good, M.H. Spitzer et al., "Automated mapping of phenotype space with single-cell data," Nature Methods, vol. 13, no. 6, pp. 493-496, 2016.
20. A.M. Gizinski, D.A. Fox, "T cell subsets and their role in the pathogenesis of rheumatic disease," Current Opinion Rheumatology, vol. 26, no. 2, pp. 204-210, 2014.
21. C. Scheinecker, L. Goschl, M. Bonelli, "Treg cells in health and autoimmune diseases: New insights from single cell analysis," Journal of Autoimmunity, vol. 110, pp. 102376, 2020.
22. I. Cammarata, C. Martire, A. Citro et al., "Counter-regulation of regulatory T cells by autoreactive CD8(+) T cells in rheumatoid arthritis," Journal of Autoimmunity, vol. 99, pp. 81-97, 2019.
23. H.Y. Wu, S. Han, B.B. Wu et al., "Single-cell mass cytometry reveals in vivo immunological response to surgical biomaterials," Applied Materials Today, vol. 16, pp. 169-178, 2019.
24. S. Yamanouchi, K. Kuwahara, A. Sakata et al., "A T cell activation antigen, Ly6C, induced on CD4^+^ Th1 cells mediates an inhibitory signal for secretion of IL-2 and proliferation in peripheral immune responses," European Journal of Immunology, vol. 28, no. 2, pp. 696-707, 1998.
25. N.C. Peters, A.J. Pagan, P.G. Lawyer et al., "Chronic parasitic infection maintains high frequencies of short-lived Ly6C+CD4+ effector T cells that are required for protection against re-infection," PLoS Pathogens, vol. 10, no. 12, pp. e1004538, 2014.
26. M. Tham, G.R. Schlor, D. Yerly, et al., Reduced pro-inflammatory profile of gammadeltaT cells in pregnant patients with rheumatoid arthritis, Arthritis Research & Therapy, vol. 18, no. 1, pp. 26-35, 2016.
27. P. Khandelwal, A. Lane, E. Owsley et al., "Peripheral blood expansion of CD38 bright CD8^+^ effector memory T-cells predicts acute graft versus host disease with a diagnostic accuracy of 87%," Biology of Blood and Marrow Transplantation, vol. 21, no. 7, pp. S99-S101, 2015.
28. L. Bader, S.E. Gullaksen, N. Blaser, M. Brun, G.H. Bringeland, A. Sulen, C.G. Gjesdal, C. Vedeler, S. Gavasso, “Candidate Markers for Stratification and Classification in Rheumatoid Arthritis, Front Immunol”, vol.10 ,pp.1488,2019.
29. U. Baddack-Werncke, M. Busch-Dienstfertig, S. Gonzalez-Rodriguez, S.C. Maddila, J. Grobe, M. Lipp, C. Stein, G. Muller, Cytotoxic T “cells modulate inflammation and endogenous opioid analgesia in chronic arthritis”, Journal of Neuroinflammation, vol.14,pp. 30, 2017.
30. F. Zhang, K. Wei, K. Slowikowski, et al., “Defining inflammatory cell states in rheumatoid arthritis joint synovial tissues by integrating single-cell transcriptomics and mass cytometry”, Nature Immunology, vol.20, pp. 928-942, 2019.
31. L.Q. Wang, Y. Wang, R. Wang, R.X. Zhao, Y. Meng, L.L. Ren, D. Cao, X.L. Sun, X.W. Zhang, “Changes and clinical significance of peripheral blood CD8(+)CD25(+)T cells in rheumatoid arthritis patients”, Zhonghua Yi Xue Za Zhi, vol.100, pp. 1557-1561, 2020.
32. S. Khan, J.D. Greenberg, N. Bhardwaj, "Dendritic cells as targets for therapy in rheumatoid arthritis," Nature Reviews Rheumatology, vol. 5, no. 10, pp. 566-571, 2009.
33. M. Alahdal, H. Zhang, R. Huang, W. Sun, Z. Deng, L. Duan, H. Ouyang, D. Wang, “Potential efficacy of dendritic cell immunomodulation in the treatment of osteoarthritis”, Rheumatology (Oxford), vol.60, pp. 507-517, 2021.
34. G. Reynolds, J.R. Gibbon, A.G. Pratt, M.J. Wood, D. Coady, G. Raftery, A.R. Lorenzi, A. Gray, A. Filer, C.D. Buckley, M.A. Haniffa, J.D. Isaacs, C.M. Hilkens, “Synovial CD4+ T-cell-derived GM-CSF supports the differentiation of an inflammatory dendritic cell population in rheumatoid arthritis”, Annals of the Rheumatic Diseases, vol. 75, pp. 899-907, 2016.
35. L. Saveanu, O. Carroll, M. Weimershaus, P. Guermonprez, E. Firat, V. Lindo, F. Greer, J. Davoust, R. Kratzer, S.R. Keller, G. Niedermann, P. van Endert, “IRAP identifies an endosomal compartment required for MHC class I cross-presentation”, Science, vol.325, pp. 213-217, 2009.
36. C. Wang, X. Liu, Z. Li, Y. Chai, Y. Jiang, Q. Wang, Y. Ji, Z. Zhu, Y. Wan, Z. Yuan, Z. Chang, M. Zhang, “CD8(+)NKT-like cells regulate the immune response by killing antigen-bearing DCs”, Scientific Reports, vol. 5, pp. 14124, 2015.
37. L.G. van Baarsen, M.J. de Hair, T.H. Ramwadhdoebe, I.J. Zijlstra, M. Maas, D.M. Gerlag, P.P. Tak, “The cellular composition of lymph nodes in the earliest phase of inflammatory arthritis”, Ann Rheum Dis, vol. 72, pp. 1420-1424, 2013.
38. C. Croia, B. Serafini, M. Bombardieri, S. Kelly, F. Humby, M. Severa, F. Rizzo, E.M. Coccia, P. Migliorini, F. Aloisi, C. Pitzalis, “Epstein-Barr virus persistence and infection of autoreactive plasma cells in synovial lymphoid structures in rheumatoid arthritis”, Ann Rheum Dis, vol. 72, pp. 1559-1568, 2013.
39. H. Carvalheiro, C. Duarte, S. Silva-Cardoso, J.A. da Silva, M.M. Souto-Carneiro, “CD8+ T cell profiles in patients with rheumatoid arthritis and their relationship to disease activity”, Arthritis Rheumatol, vol. 67, pp. 363-371, 2015.
40. M. Merad, P. Sathe, J. Helft, J. Miller, A. Mortha, “The dendritic cell lineage: ontogeny and function of dendritic cells and their subsets in the steady state and the inflamed setting”, Annual Review Immunology, vol. 31, pp. 563-604, 2013.
41. P.C. Taylor, A.J. Holman, “Rheumatoid arthritis and the emergence of immuno-autonomics”, Rheumatology (Oxford), vol. 58, pp. 2079-2080, 2019.
42. J.C. Gigliotti, L. Huang, H. Ye, A. Bajwa, K. Chattrabhuti, S. Lee, A.L. Klibanov, K. Kalantari, D.L. Rosin, M.D. Okusa, “Ultrasound prevents renal ischemia-reperfusion injury by stimulating the splenic cholinergic anti-inflammatory pathway”, Journal of the American Society of Nephrology, vol. 24, pp. 1451-1460,2013.
43. X. Qiu, Q. Mao, Y. Tang et al., "Reversed graph embedding resolves complex single-cell trajectories," Nature Methods, vol. 14, no. 10, pp. 979-982, 2017.
44. M. Efremova, M. Vento-Tormo, S.A. Teichmann et al., "CellPhoneDB: inferring cell-cell communication from combined expression of multi-subunit ligand-receptor complexes," Nature Protocols, vol. 15, no. 4, pp. 1484-1506, 2020.
45. M.P. Murphy, L.S. Koepke, M.T. Lopez, X. Tong, T.H. Ambrosi, G.S. Gulati, O. Marecic, Y. Wang, R.C. Ransom, M.Y. Hoover, H. Steininger, L. Zhao, M.P. Walkiewicz, N. Quarto, B. Levi, D.C. Wan, I.L. Weissman, S.B. Goodman, F. Yang, M.T. Longaker, C.K.F. Chan, "Articular cartilage regeneration by activated skeletal stem cells, " Nature Medicine, vol. 26, pp. 1583-1592, 2020.
46. D. Amarasekara, H. Yun, S. Kim, N. Lee, H. Kim, J. Rho, "Regulation of osteoclast differentiation by cytokine networks," Immune Network, vol. 18, no. 1, pp. e8, 2018.
47. K.S. Boles, W. Barchet, T. Diacovo, M. Cella, M. Colonna, "The tumor suppressor TSLC1/NECL-2 triggers NK-cell and CD8^+^ T-cell responses through the cell-surface receptor CRTAM," Blood, vol. 106, no. 3, pp. 779-786, 2005.
48. M.A. van Maanen, M.J. Vervoordeldonk, P.P. Tak, "The cholinergic anti-inflammatory pathway: towards innovative treatment of rheumatoid arthritis," Nature Reviews Rheumatology, vol. 5, no. 4, pp. 229-232, 2009.
49. S.I. Koizumi, D. Sasaki, T.H. Hsieh et al., "JunB regulates homeostasis and suppressive functions of effector regulatory T cells," Nature Communication, vol. 9, no. 1, pp. 5344, 2018.
50. E. Sebzda, Z. Zou, J.S. Lee et al., "Transcription factor KLF2 regulates the migration of naïve T cells by restricting chemokine receptor expression patterns," Nature Immunology, vol. 9, no. 3, pp. 292-300, 2008.
51. S. Wang, R. Song, Z. Wang et al., "S100A8/A9 in Inflammation," Frontiers in Immunology, vol. 11, no. 9, pp. 1298, 2018.
52. D.A. Liebermann, B. Gregory, B. Hoffman, "AP-1 (Fos/Jun) transcription factors in hematopoietic differentiation and apoptosis," International Journal of Oncology, vol. 12, no. 3, pp. 685-700, 1998.
53. D. Passeri, A. Marcucci, G. Rizzo et al., "Btg2 enhances retinoic acid-induced differentiation by modulating histone H4 methylation and acetylation," Molecular and Cellular Biology, vol. 26, no. 13, pp. 5023-5032, 2006.
54. R. Acin-Perez, M.P. Bayona-Bafaluy, M. Bueno et al., "An intragenic suppressor in the cytochrome c oxidase I gene of mouse mitochondrial DNA," Human Molecular Genetics, vol. 12, no. 3, pp. 329-339, 2003.
55. X. Zhou, W.J. Liao, J.M. Liao et al., "Ribosomal proteins: functions beyond the ribosome," Journal of Molecular Cell Biology, vol. 7, no. 2, pp. 92-104, 2015.
56. P. Rwei, C.S. Alex Gong, L.J. Luo et al., "In vitro investigation of ultrasound-induced oxidative stress on human lens epithelial cells," Biochemical and Biophysical Research Communications, vol. 482, no. 4, pp. 954-960, 2017.
57. T. Bian, W. Meng, M. Qiu et al., "Noninvasive ultrasound stimulation of ventral tegmental area induces reanimation from general anaesthesia in mice," Research, vol. 2021, pp. 2674692, 2021 (2021).
58. H. Zhou, L. Niu, L. Meng et al., "Noninvasive ultrasound deep brain stimulation for the treatment of parkinson's disease model mouse," Research, vol. 2019, pp. 1748489, 2019 .
59. K.M. Pietrosimone, M. Jin, B. Poston et al., "Collagen-induced arthritis: a model for murine autoimmune arthritis," Bio-Protocol, vol. 5, no. 20, pp. e1626, 2015.
60. L. Stangenberg, D. Burzyn, B.A. Binstadt et al., "Denervation protects limbs from inflammatory arthritis via an impact on the microvasculature," Proceedings of the National Academy of Sciences of the United States of America, vol. 111, no. 31, pp. 11419-11424, 2014.
61. S. Chevrier, J.H. Levine, V.R.T. Zanotelli et al., "An immune atlas of clear cell renal cell carcinoma," Cell, vol. 169, no. 4, pp. 736-749, 2017.
62. G. Han, M.H. Spitzer, S.C. Bendall et al., "Metal-isotope-tagged monoclonal antibodies for high-dimensional mass cytometry," Nature Protocol, vol. 13, no. 10, pp. 2121-2148, 2018.
63. Y. Lavin, S. Kobayashi, A. Leader et al., "Innate immune landscape in early lung adenocarcinoma by paired single-cell analyses," Cell, vol. 169, no. 4, pp. 750-765, 2017.
64. Y.M. Chen, P. Lin, J.Q. He et al., "Combination of the manifold dimensionality reduction methods with least squares support vector machines for classifying the species of sorghum seeds," Scientific Reports, vol. 6, pp. 19917, 2016.
65. C. Trapnell, D. Cacchiarelli, J. Grimsby et al., "The dynamics and regulators of cell fate decisions are revealed by pseudotemporal ordering of single cells," Nature Biotechnology, vol.32, no. 4, pp. 381-386, 2014.
66. I.A. Udalova, A. Mantovani, M. Feldmann, "Macrophage heterogeneity in the context of rheumatoid arthritis," Nature Reviews Rheumatology, vol.12, no. 8, pp. 472-485, 2016.
67. X. Zhang, N. Olsen, S.G. Zheng, "The progress and prospect of regulatory T cells in autoimmune diseases, Journal of Autoimmunity," vol. 111, pp. 102461, 2020.
68. A.K. Rana, Y. Li, Q. Dang et al., "Monocytes in rheumatoid arthritis: circulating precursors of macrophages and osteoclasts and, their heterogeneity and plasticity role in RA pathogenesis," International Immunopharmacology, vol. 65, pp. 348-359, 2018.
